# The role of ER-associated degradation and ER-phagy in health and disease

**DOI:** 10.1038/s41392-025-02501-7

**Published:** 2026-01-05

**Authors:** Young Joo Jeon, Ze’ev A. Ronai

**Affiliations:** 1 Department of Biochemistry, Chungnam National University College of Medicine, Daejeon, 35015 Republic of Korea; 2 Brain Korea 21 FOUR Project for Medical Science, Chungnam National University, Daejeon, Republic of Korea; 3 Department of Medical Science, Chungnam National University College of Medicine, Daejeon, 35015 Republic of Korea; 4 Translational Research Institute, Jim and Eleanor Randall Department of Surgery and the Department of Biomedical Sciences, Cedars Sinai Medical Center, Los Angeles, CA 90048 USA

**Keywords:** Biochemistry, Cell biology

## Abstract

The endoplasmic reticulum (ER) is a major cellular organelle for the synthesis and folding of secretory and transmembrane proteins, whose proper function underpins organellar homeostasis, proper tissue function, and organismal physiology. Protein quality control (PQC) systems at the ER include the unfolded protein response (UPR), ER-associated degradation (ERAD), and ER-phagy, which monitor ER homeostasis and contribute to protein refolding, sequestration, or degradation. ERAD prevents the accumulation of misfolded or orphan proteins that would otherwise be toxic. By controlling the degradation of these proteins, ERAD performs a core function in governing adaptation to proteotoxic stress. ERAD also regulates the abundance of folding-competent proteins as a means to fine-tune key physiological processes. Among its complex regulatory activities, ERAD controls cellular processes such as lipid homeostasis, calcium flux, and cell fate decisions, which are all required for the maintenance of organelle homeostasis. Highlighting its importance, dysregulation of ERAD often results in devastating diseases. Here, we discuss the molecular and mechanistic understanding of protein quality and quantity control by ERAD and its interface with ER-phagy, as well as other cellular stress programs. The implications of ERAD and its associated regulatory arms for cellular homeostasis, its effects on health and disease, and current therapeutic approaches are discussed.

## Introduction

The well-being and functionality of the proteome, often referred to as proteostasis, ensures tissue integrity and organismal health.^1,2^ These activities are constantly challenged by intrinsic and extrinsic perturbations, including increased protein synthesis, ER-Ca^2+^ depletion, dysregulated redox homeostasis, energy deprivation, hypoglycemia, hypoxia, and inflammatory stimuli, resulting in improper protein conformation and misfolding, often referred to as nonnative or aberrant conformation.^2,3^ Impaired refolding of either unfolded or misfolded proteins through chaperones, or diminished degradation through proteasomes or lysosomes, results in the formation and accumulation of protein aggregates, exemplifying proteostasis dysfunction. The latter represents a compromised cell’s ability to overcome intrinsic and extrinsic perturbations.^4,5^ Deleterious cellular effects triggered by proteostatic dysfunction are referred to as proteotoxicity, which is implicated in various human pathologies.^6^

**Fig. 1:**
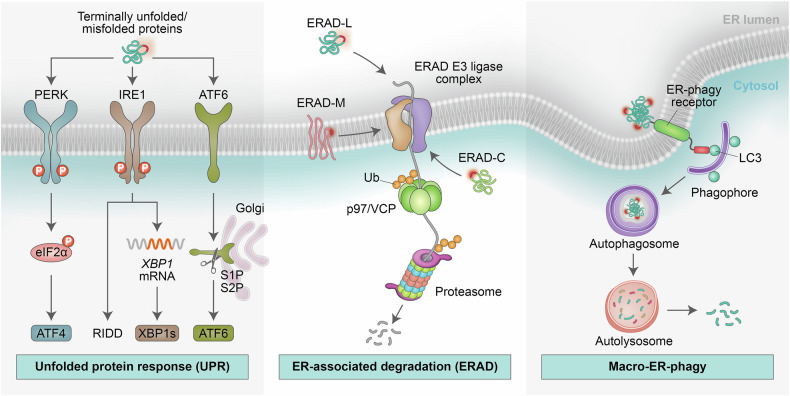
ER protein quality control (PQC) systems. *Unfolded protein response (UPR)*: Under proteotoxic stress in the ER, cells activate the UPR to increase protein-folding capacity, increase PQC, and facilitate the degradation of terminally unfolded/misfolded proteins. UPR initiation relies on three key stress sensors located on the ER membrane: inositol-requiring protein 1 (IRE1) α and β, activating transcription factor 6 (ATF6) α and β, and protein kinase RNA (PKR)-like ER kinase (PERK). PERK phosphorylates eIF2α, thereby decreasing global protein synthesis, whereas eIF2α phosphorylation leads to selective ATF4 expression. IRE1, which functions as both a serine/threonine kinase and an endoribonuclease (RNase), is activated via autotransphosphorylation of its RNase domain. IRE1 undergoes dimerization and/or oligomerization, which subsequently triggers its transphosphorylation and activation, whose changes are required for nuclease activity. IRE1 splices mRNA that encodes X-box binding protein-1 (XBP1) to generate XBP1s. The RNase activity of IRE1 also regulates the stability of RNA via regulated IRE1-dependent decay (RIDD). ATF6, a transmembrane transcription factor, is processed in the Golgi apparatus, where it produces its cytosolic fragment (ATF6f), which serves as a transcription factor. *ER-associated degradation (ERAD)* (see text for details): Terminally unfolded/misfolded proteins are retained in the ER by recognition factors that trigger the steps required for essential ubiquitin–proteasome system (UPS)-mediated ERAD, which include translocation and extraction of proteins to the cytoplasm, the location at which ubiquitination takes place. *Macro-ER-phagy:* Macro-ER-phagy is dependent on ER-phagy receptors, which contain LC3-interacting regions (LIRs) that enable binding to the LC3-decorated isolation membrane (phagophore). The LC3-decorated autophagosome engulfs terminally unfolded/misfolded proteins and protein aggregates and then delivers them to the lysosome for degradation

One-third of the mammalian proteome is directed to the ER, where proteins undergo synthesis, posttranslational modification, folding, and assembly prior to their transport to various cellular organelles and the plasma membrane or their release from cells via the secretory pathway.^7^ Notably, a subset of newly synthesized polypeptides in the ER fails to reach a native conformation, engaging cellular processes, including protein quality control (PQC), which includes the unfolded protein response (UPR) as part of ER-associated degradation (ERAD) and ER-phagy. These well-coordinated cellular processes continuously monitor organellar homeostasis and channel misfolded proteins toward refolding, sequestration, or degradation (Fig. 1).^3,8–12^ The coordinated UPR response includes cytoplasmic-to-nuclear signaling designed to increase the protein-folding capacity of the ER, reduce protein input into the ER, and activate ERAD and, if needed, autophagy. ERAD and ER-phagy are involved in the degradation of misfolded proteins and protein aggregates. The ERAD functions as part of PQC via the ubiquitin–proteasome system (UPS).^13^ ER-phagy utilizes the core autophagy machinery to eliminate ERAD-resistant misfolded proteins that are processed by the lysosome.^9,12,14–16^

During eukaryotic evolution, complex ERAD pathways have emerged to maintain proteome integrity and functionality.^17^ While ERAD was previously thought to be associated with PQC, which safeguards protein maturation, recent studies have demonstrated that ERAD also regulates the abundance of folding-competent proteins as a means to fine-tune key physiological processes, suggesting that ERAD constitutively functions to ensure both protein fidelity and abundance.^18^ Support for this system’s importance emerges from adverse outcomes and even lethality when ERAD components are impaired embryonically.^8^ Therefore, understanding the mechanisms underlying ERAD activity and its physiological relevance is an important area of increasing research interest. In this review, we discuss the molecular and mechanistic understanding of protein quality and quantity control by ERAD and its integration with ER-phagy, as with other cellular stress programs. The physiological implications of recent advances in ERAD research and recent therapies that address ERAD-related diseases are also discussed.

## Erad control of protein quality and quantity via the ubiquitin-proteasome system (UPS)

**Fig. 2:**
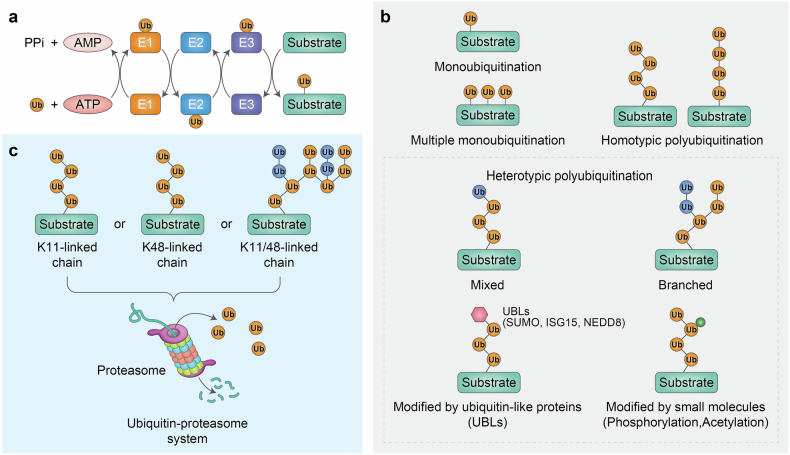
Ubiquitination, ubiquitin code, and ubiquitin–proteasome system (UPS). **a** Ubiquitination, a posttranslational modification of a substrate protein by ubiquitin, is a multistep process carried out by E1 ubiquitin-activating enzymes, E2 ubiquitin-conjugating enzymes, and E3 ubiquitin ligases, which successively activate, conjugate, and ligate ubiquitin to the substrate protein. Initially, E1 activates ubiquitin in an ATP-dependent manner. The activated ubiquitin is subsequently transferred to E2 and ultimately ligated to a lysine residue on the substrate protein by E3. **b** Monoubiquitination is the simplest type of ubiquitination. Additional ubiquitins are conjugated through the same processes to generate polyubiquitin chains. In homotypic polyubiquitination, one ubiquitin can be conjugated to another either through one of its seven lysine residues (Lys6, Lys11, Lys27, Lys29, Lys33, Lys48, and Lys63) or through its N-terminal methionine. In heterotypic polyubiquitination, ubiquitin molecules can be conjugated via two or more distinct linkage types during a single polymerization process, forming a mixed polyubiquitin chain. A single ubiquitin can undergo modifications at multiple sites, leading to the formation of a branched polyubiquitin chain. Additionally, during polyubiquitination, ubiquitin can be attached to ubiquitin-like proteins (UBLs), including ISG15, SUMO, and NEDD8. Ubiquitin can also be phosphorylated or acetylated. **c** Proteins designated for proteolytic degradation by the 26S proteasome are marked with a polyubiquitin chain to form a ubiquitin code that acts as a signal for the proteasome. The polyubiquitin chains target the substrate protein for proteasomal degradation. Homotypic K48-linked or K11-linked polyubiquitin chains mediate proteasomal degradation. Heterotypic K11/K48-linked branched polyubiquitin chains that could ensure an increase in the local ubiquitin concentration on the substrates enhance proteasomal degradation (see text for details)

Ubiquitination is a sequential process orchestrated by core E1 ubiquitin-activating enzymes, general E2 ubiquitin-conjugating enzymes, and selective E3 ubiquitin ligases, which, in concert, conjugate ubiquitin moieties in select topologies to a substrate (Fig. 2a).^19^ This coordinated process is controlled by posttranslational modification of the substrates, such as the ubiquitination machinery components, which dictates the type (mono- or poly-) of ubiquitination.^20^ Ubiquitin harbors eight sites available for ubiquitination, including seven internal lysine (Lys, K) residues (K6, K11, K27, K29, K33, K48, and K63) and the α-amino group of the N-terminal methionine (M1), which contribute to the formation of distinct homotypic polyubiquitin chains and their unique topologies. The generation of heterotypic polyubiquitin chains occurs via the assembly of mixed ubiquitin chains or branched ubiquitin chains, with ubiquitin being modified at multiple sites.^21–23^ The architecture of polyubiquitin chains is primarily dictated by E2, as by a number of parameters on the substrate itself.^24^ Intriguingly, the architecture of ubiquitination encompasses monoubiquitination, multiple monoubiquitination, as well as eight distinct types of homotypic polyubiquitin linkages, and various forms of heterotypic polyubiquitin linkages. Each of these combinations governs substrate fate which serve to maintain cellular homeostasis (Fig. 2b).^21,24–26^ For example, monoubiquitination, which can take place at multiple lysine residues (multiple monoubiquitination), regulates various processes, such as allosteric regulation, complex formation, endocytosis, protein recognition, and even proteasome-mediated degradation.^20^ The formation of diverse polyubiquitin chains has been the topic of intense studies, which point to several interesting observations. K63-linked polyubiquitin chains have been shown to regulate cellular processes, including protein trafficking, DNA repair, autophagy, and signal transduction, whereas K48-linked polyubiquitin chains are linked with proteasomal degradation.^27–29^ K6-linked polyubiquitination has been associated with mitophagy,^30–32^ whereas K11-linked polyubiquitin chains are associated with ERAD.^22,33^ Interestingly, heterotypic branched chains were also identified, exemplified by the K11/K48-linked polyubiquitin chains, which act as priority signals for the proteasome, directing aggregation-prone and cytotoxic proteins toward proteasomal degradation.^33,34^

The UPS serves as the primary cellular degradation system via the 26S proteasome. Proteins targeted to the 26S proteasome undergo ubiquitination through sequential enzymatic reactions involving E1, E2, and E3 enzymes, thereby generating the ubiquitin code that serves as a signal for proteasome-mediated degradation (Fig. 2c). The UPS mediates the selective degradation of proteins that participate in diverse cellular processes, thereby maintaining a fine balance of cellular activities. It also serves as the primary site for the clearance of misfolded or damaged proteins.^35^ The 26S proteasome comprises two main components: 19S regulatory and 20S core particles. The 19S regulatory particle recognizes ubiquitinated proteins and then deubiquitinates, unfolds, and translocates the substrate proteins to the catalytic 20S core, where enzyme-catalyzed cleavage of the substrate protein to its peptides and amino acids takes place.^35^

**Fig. 3:**
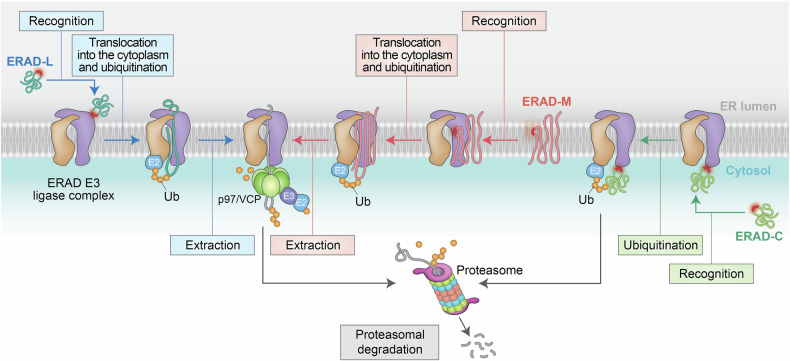
ER-associated degradation (ERAD). General steps associated with ERAD (see text for details). ERAD substrates are categorized as ERAD-L, ERAD-M, or ERAD-C, according to where the misfolded domain (degron) is located. Once ERAD substrates are recognized and ubiquitinated by ERAD E3 ubiquitin ligases embedded in the ER membrane and cytoplasmic E1 and E2 enzymes, they are extracted from the ER to the cytosol by the p97/VCP ATPase. The extracted substrates are then trimmed and reubiquitinated before proteasomal degradation. The aggregation of extracted substrates is inhibited by ubiquitin-binding proteins, which additionally support the subsequent transfer of ERAD substrates to the proteasome. ERAD-C directly routes proteins to the proteasome in the absence of support from p97/VCP

The high burden of protein synthesis imposed on the ER led to the initial recognition of the existence and importance of ERAD.^36^ Initially, described as a site for the removal of unassembled, “orphan” subunits of protein complexes from the ER,^37,38^ ERAD was subsequently linked with the UPS.^39–42^ This process requires an active escort mechanism, whereby the AAA+ ATPase p97/valosin-containing protein (VCP) (known as Cdc48 in yeasts) extracts misfolded, damaged, or improperly synthesized proteins from the ER to enable their recognition and processing by ER-dedicated E3 ubiquitin ligases, which mark them for degradation by the 26S proteasome.^13,18^ Multiple parallel pathways contribute to the recognition and translocation of different secretory and membrane substrates from the ER into the cytoplasm. ERAD substrates with folding problems or degradation signals (degrons) in either the ER lumen (L), the membrane (M), or the cytoplasmic domain (C) are targeted by the ERAD branches referred to as ERAD-L, ERAD-M, and ERAD-C, respectively, for processing by dedicated E3 ubiquitin ligases and subsequently by proteasomes (Fig. 3).^36^

### ERAD controls the switch from folding to degradation

The coordinated action of molecular chaperones in the ER is essential for the decision to prevent proteotoxicity by restoring folding or by promoting degradation. Several components that participate in this process include carbohydrate-binding lectins, calnexin and calreticulin, binding immunoglobulin protein (BiP) and its cofactors, heat shock protein 70 (HSP70), and oxidative folding enzymes. Triage decisions relevant to not only whether misfolded proteins are reparable for folding but also whether protein aggregates can be solubilized determine the fate of the misfolded and/or aggregated protein species that will be subjected to the repair pathway (i.e., refolding) or be removed (i.e., degraded) to maintain proper proteostasis.^3,43,44^

A long-standing question relates to the assessment of protein status and fate. Specifically, how ERAD chaperones assess whether a client protein exhibits slow folding kinetics and could be protected from degradation or should be degraded as it is terminally misfolded is unknown. Some chaperones dedicated to the folding of nascent polypeptides act as recognition components to signal pro-degradation.^45,46^ The *N*-linked glycan, which is attached to an Asn residue in the consensus Asn-X-Ser/Thr (X: any amino acid except Pro) sequence of a protein, originally comprises three glucoses, nine mannoses, and two *N*-acetylglucosamines and was designated Glc_3_Man_9_GlcNAc_2_. Glycoproteins in the ER possess derivatives of the *N*-linked glycan moiety, which is achieved by *N*-glycan trimming processes.^47,48^ Glucosidases I and II in the ER progressively remove glucose residues from the *N*-glycans of newly synthesized glycoproteins to generate Glc_1_Man_9_GlcNAc_2_, which is detected by the calnexin/calreticulin cycle and serves as a signal to promote glycoprotein folding. Glucosidase II further processes Glc_1_Man_9_GlcNAc_2_ to produce Man_9_GlcNAc_2_. At this point, two paths are possible. First, glycoproteins that are not yet correctly folded are reglycosylated and re-engaged by the calnexin/calreticulin cycle. Second, glycoproteins that are unable to fold into their native three-dimensional structure are subject to the initial removal of mannose to be Man_8_GlcNAc_2_, which leads to their permanent exit from the calnexin/calreticulin cycle. Particular branch structures of the *N*-glycan, Man_7_GlcNAc_2_, Man_6_GlcNAc_2_, and Man_5_GlcNAc_2_, all of which expose a terminal α1,6-linked mannosyl residue and prevent reglycosylation, become signals for degradation via ERAD, directing a misfolded protein from pro-folding to pro-degradation.^47,48^ Man_7_−, Man_6_−, and Man_5_-containing glycans are generated through the stepwise action of ER degradation-enhancing mannosidase 2 (EDEM2), which is followed by EDEM1/EDEM3.^48^ The modified glycan is detected by another ER-resident lectin, osteosarcoma amplified 9 (OS9), which binds to luminal portions of the retrotranslocation channel.^49^ The interaction of ER-resident DnaJ/JDP (ERdj) 5 with EDEMs and OS9 promotes further glycan processing and breaks disulfide bonds of the misfolded glycoprotein, thereby enabling the misfolded glycoprotein to translocate from the ER into the cytoplasm.^50,51^ Nonglycosylated, misfolded proteins may be targeted for degradation via a mechanism involving substrate transfer from BiP to ERdjs.^52–54^ In cooperation with its cofactors ERdjs, BiP contributes to the translocation of nascent polypeptides into the ER lumen,^55,56^ the folding and assembly of nascent polypeptides,^57^ the regulation of the UPR,^58,59^ and the selection of ERAD substrates.^53,60^ While ERdj3 and ERdj6 function as cofactors of BiP, enabling nascent polypeptides to fold,^61^ ERdj4 and ERdj5 assist BiP in ERAD. ERdj4 binds to aggregation-prone proteins and maintains their solubility in ERAD.^62^ ERjd4 also functions to recruit ER substrates to ERAD sites.^52^ Additionally, ERdj5 catalyzes disulfide bond reduction and unfolds proteins, ensuring efficient substrate translocation into the cytoplasm for ERAD.^53,63^ ERdj5 also disaggregates proteins to target them for ERAD, a process enhanced by BiP.^54^ Another component involved in this process is glucose-regulated protein 170 (GRP170), which enhances client protein degradation when overexpressed.^64,65^ Notably, ERdj4, ERdj5, and GRP170 selectively recognize a distinct type of rarer peptide that contains high levels of aromatic residues and tends to form aggregates, which directs the client protein from pro-folding to pro-degradation.^66^ While BiP and GRP94 regulate the switch from pro-folding to pro-degradation,^44^ GRP94 maintains the solubility of aggregation-prone proteins to enable their translocation into the cytoplasm,^67,68^ although GRP94 has a weaker interaction with the ERAD machinery than BiP does.

### Substrate recognition: routes that recognize and distinguish substrates

**Fig. 4:**
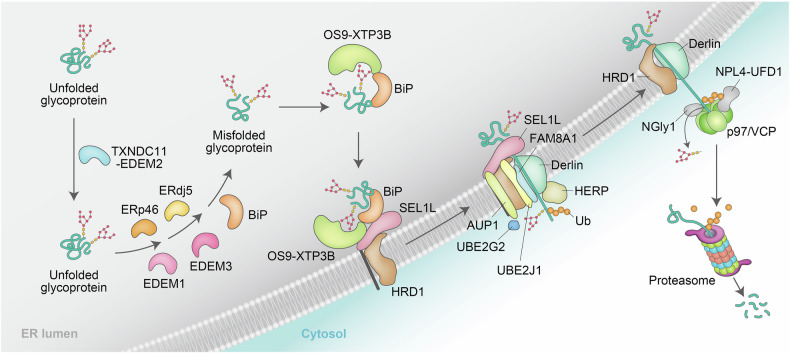
Proposed ERAD by the SEL1L–HRD1 complex. Misfolded luminal glycoproteins that fail to achieve proper three-dimensional structures are initially recognized by EDEM2 and TXNDC11 (TXNDC11–EDEM2) for the removal of the outermost mannose from Man_9_GlcNAc_2_, generating Man_8_GlcNAc_2_. These proteins are subsequently processed by EDEM3 or EDEM1, which further trim mannoses to generate specific branched structures of *N*-glycans, Man_7_GlcNAc_2_, Man_6_GlcNAc_2_, or Man_5_GlcNAc_2_. The *N*-glycan exposing an α1,6-linked mannosyl residue is captured by OS9 and XTP3B (OS9–XTP3B), which interact with BiP to engage PQC degrons and form the HRD1 E3 complex by associating with SEL1L. Once bound to OS9–XTP3B, the substrates align with a proteinaceous channel in the ER membrane, likely composed of TMDs of Derlin, HRD1, and HERP, and are translocated from the ER to the cytosol. AUP1 is required for the ER membrane recruitment of UBE2G2. FAM8A1 plays a role in the formation of the HRD1 complex and in the presentation of substrates to the ubiquitination machinery. Substrates polyubiquitinated by the SEL1L–HRD1 complex with the aid of UBE2J1 and UBE2G2 are extracted by p97/VCP. NPL4–UFD1 interacts with p97/VCP and captures substrates via its Lys48-linked polyubiquitin signature, promoting the unfolding of ubiquitin within the chain as it inserts into a groove in NPL4–UFD1. NGly1 might be recruited to p97/VCP and remove *N*-linked oligosaccharides after substrate translocation to the cytoplasm prior to engagement by p97/VCP. The extracted substrates are then directed to the proteasome for degradation (see the text for details)

ERAD follows three distinct, albeit overlapping, pathways with different substrate selectivities. These three proteins differentially recognize ERAD-L, ERAD-M, or ERAD-C substrates harboring a degron within the ER lumen (L), lipid bilayer (M), or cytoplasm (C), respectively.^36^ *N*-glycans are important for substrate selection in ERAD-L. Fruitless repair attempts after numerous folding cycles prolong the presence of immature substrates in the ER lumen, resulting in elevated exposure of *N*-linked glycans to ER-resident mannosidases and glucosidases. Disulfide bonding is a conformational obstacle to substrate translocation.^69^ EDEMs interact with redox factors, including protein disulfide–isomerase (PDI), thioredoxin domain-containing 11 (TXNDC11), ER-resident protein 46 (ERp46), and ERdj5, and form complexes that facilitate mannose trimming activity via EDEMs.^70–72^ ERdj5 and TXNDC11 cleave disulfide bonds of luminal substrates and accelerate ERAD via physical and functional associations with EDEMs.^53,73^ The disulfide bond reduction and irreparable trimming of *N*-linked glycans generate degrons selectively modified with glycans such as Man_7_GlcNAc_2_, Man_6_GlcNAc_2_, or Man_5_GlcNAc_2_, which prevents substrate re-engagement with calnexin or calreticulin for nonproductive folding cycles.^73^ The ER lectins OS9 and XTP3B preferentially recognize glycosylated degrons within substrates and interact with BiP to engage PQC degrons,^1^ which are hydrophobic polypeptide segments exposed on the substrate surface.^67,74–78^ Interactions of the suppressor/enhancer of lin12-like (SEL1L) with several chaperones, including BiP, homoCys-responsive ER-resident protein (HERP), OS9, XTP3B (endoplasmic reticulum lectin 1 (ERLEC1)), PDI, EDEMs, and ERdj5, have been implicated in substrate recognition.^78,79^ SEL1L contributes to substrate recruitment via its interaction with OS9 and XTP3B, which recognize Man_7_−, Man_6_−, and Man_5_-containing glycans and form a luminal surveillance complex that captures and delivers substrates to the ERAD E3 ubiquitin ligase hydroxymethylglutaryl reductase degradation protein 1 (HRD1) (Fig. 4).^80–84^ ERAD-L without *N*-linked oligosaccharides and consequently without glycosylated degrons yet associates with chaperones (BiP, ERdj5, and EDEM1) and HERP for delivery to SEL1L-HRD1.^53,70,85–87^ SEL1L was shown to be required for the recruitment of UBE2J1 and Derlin to HRD1, suggesting that the interaction between SEL1L and HRD1 is essential for establishing a functional HRD1 ERAD complex.^88^ The engagement of lipids in ERAD is exemplified by the ER-resident intramembrane rhomboid protease RHBDL4, which is associated with the ER lipid raft-associated protein 1 (ERLIN1)-ERLIN2 complex and catalyzes the cleavage of aggregation-prone proteins, targeting proteolytic fragments for ERAD.^89^

The nature of the degrons (the hydrophobicity and length of their transmembrane domains (TMDs)) present in the ER membrane and their position relative to the ER membrane determine membrane substrate recognition, access to ERAD E3 ubiquitin ligases, and the disposal route. Integral membrane substrates harboring glycosylated degrons exposed to the ER lumen laterally diffuse within the ER membrane, engaging ER lectins and being directed toward HRD1.^90–92^ Single-pass membrane proteins that contain extensive luminal domains (i.e., major histocompatibility complex (MHC) class I and cluster of differentiation 147 (CD147)) depend on HRD1 for substrate recognition in ERAD. In contrast, integral membrane substrates with degrons present in TMDs or within cytoplasmic domains are recognized by HRD1 in an ER lectin-independent manner or by other ERAD E3 ubiquitin ligases equivalently or preferentially. 3-Hydroxy-3-methyl-glutaryl-coenzyme A reductase (HMGCR) is recruited for ERAD via insulin-induced genes (INSIGs) and exhibits preferential preference for certain ERAD E3 ubiquitin ligases, exemplified by glycoprotein 78 (gp78), chromosome 8 translocation in renal carcinoma (TRC8), and ring finger protein 145 (RNF145).^93,94^ Tail-anchored membrane proteins with minimal exposure to the ER lumen are recognized by membrane-associated RING C3HC4 finger 6 (MARCHF6) following cleavage of their tail domains by signal peptide peptidase (SPP)^95–97^ to enable ubiquitination. High cholesterol dissociates squalene monooxygenase (SM) from the ER membrane, promoting recognition by MARCHF6.^98^ RNF185, an RNF5 paralog,^99^ forms an ERAD complex with membralin (MBRL, also known as transmembrane protein 259 (TMEM259)) and transmembrane and ubiquitin-like domain-containing proteins 1 and 2 (TMUB1/2) for substrate recognition. RNF185 was also found to be a part of a complex with UBE2K,^100^ with concomitant engagement of the cytosolic E3 ligase UBE3C to degrade membrane substrates. The TMD of cytochrome P450 family 51 subfamily A member 1 (CYP51A1) has been demonstrated to be dependent on RNF185 for substrate recognition,^100^ in which degrons in both the ER lumen and the TMD are needed, indicating that substrate selection is governed by multiple determinants as part of ERAD fidelity. Several cofactors function in the recognition of nonglycosylated degrons of membrane substrates within the ER lumen, such as in the selection of the disposal route. For example, ERLIN1 and ERLIN2 recognize, engage in, and deliver inositol 1,4,5-triphosphate receptor 1 (IP_3_R1) to RNF170.^101^ ERLIN2 also reportedly links the Wnt receptor protein Evi/WLS/Gpr177 with the cell growth regulator with ring finger domain 1 (CGRRF1).^102,103^ Likewise, limb region 1-like (LMBR1L) was shown to associate with and deliver Frizzled-6 to gp78.^104^

Cytoplasmic proteins are also regulated by ER-resident E3 ubiquitin ligases, although the mechanisms underlying their selection remain unclear. Among these ERAD targets are p53, peroxisome proliferator-activated receptor gamma coactivator 1-β (PGC-1β), and *N*^*6*^-adenosine methyltransferase-14 (METTL14).^105–107^ Notably, not all interactions with ER-resident E3 ubiquitin ligases result in their degradation, exemplified by p62 ubiquitination by RNF26,^108,109^ suggesting possible translocation of noncanonical ubiquitinated substrates as a possible mechanism for ERAD recognition.

### Substrate translocation: routes facilitating substrate movement from the ER to the cytoplasm

Substrate translocation across the ER membrane boundaries is an energetically unfavorable process that requires the coordination of diverse ERAD components. Recent studies have identified and characterized several factors involved in substrate translocation. Rhomboid pseudoproteases, including Derlins (yeast Der1 and Dfm1; mammalian DERL1, DERL2, and DERL3), ubiquitin-associated domain-containing protein 2 (UBAC2), and iRhoms, are implicated in substrate translocation, although none possess a proteolytic cleavage site.^110^ The rhomboid protease RHBDL4 cleaves membrane-anchored substrates through a serine‒histidine dyad and contributes to their dislocation from the membrane.^89,111,112^

Retrotranslocation, defined as the translocation of a luminal substrate from the ER to the cytosol, requires Hrd1 activity.^113,114^ Substrates recruited by Yos9 and Hrd3 are delivered to a complex containing Hrd1 and Der1, where the substrate associates with a groove in Hrd3 and a Yos9 lectin domain (on its luminal side) while its loop is inserted into the ER membrane. Accordingly, these substrates interact with the Der1 and Hrd1 lateral gates on their proximal loop domains.^113^ Retrotranslocation requires the cooperation of HRD1 with DERL2 and DERL3.^80,115^

HRD1 forms a channel, which enables the translocation of membrane substrates with various topologies.^116^ Gp78, RNF145, MARCHF6, and TRC8 possess several TMDs that may contribute to channel formation, enabling the translocation of a membrane-anchored substrate to the cytosol. In contrast, RNF5, RNF185, and CGRRF1 possess fewer TMDs, suggesting homo or hetero-oligomerization or complex formation with polytopic and nonrhomboid cofactors or with other E3 ubiquitin ligases. RNF185 forms a complex with polytopic proteins, such as TMEM259, TUMB1, and TUMB2, for dislocation.^100,117^ DERL1 forms a homotetramer with a central pore diameter sufficient to accommodate an entire substrate helix and is functionally associated with ERAD E3 ubiquitin ligases,^118–120^ suggesting that these ligases participate in the access of membrane substrates. UBAC2 binds to gp78 and ubiquitin regulatory X (UBX) domain-containing protein 8 (UBXD8), contributing to the formation of a dislocation channel.^121,122^ Intramembrane cleavage of substrate TMDs may be necessary to lower the energy barrier for efficient dislocation. RHBDL4 catalyzes the cleavage of single-spanning and polytopic membrane proteins harboring unstable transmembrane helices in a ubiquitin-dependent manner.^111,123^ The interaction of RHBDL4 with p97/VCP enables the transfer of cleaved ubiquitinated substrates, suggesting that RHBDL4 functionally links intramembrane proteolysis with dislocation in noncanonical ERAD. SPP associates with ERAD E3 ubiquitin ligases and DERL1 and internally cleaves membrane substrates to enable their dislocation.^96,97,124–126^

The lipid-thinning effects of Derlins may lower the energy requirements for substrate translocation. Examples of the latter include the Hrd1 and Der1 TMDs, which create a hydrophilic hole at the cytoplasmic side of the ER membrane, decreasing lipid bilayer thickness and facilitating substrate translocation.^113^ Dfm1 induces local membrane thinning for the extraction of membrane proteins from the ER.^127^ In contrast, the accumulation of ceramides with very long saturated acyl chains restricts the extraction of ubiquitinated substrates.^128^ Overall, lipid composition and ER membrane compaction affect both the energy barrier and the efficiency of substrate translocation.^128,129^

### Substrate ubiquitination in ERAD

During the ERAD process, ubiquitination marks substrate regions extending into the cytoplasm for recognition by the ubiquitin-dependent unfolding machinery and provides signals for substrate transport to and recognition by the 26S proteasome.^13^ To date, approximately 40 ERAD E3 ubiquitin ligases have been reported in mammals, and 3 have been reported in yeast.^18,130^ The increased number of E3 ubiquitin ligases involved in ERAD from yeast to mammals may point to the increasing complexity and diversity of ERAD that emerged during evolution. ERAD E3 ubiquitin ligases cooperate with numerous specialized factors to accommodate various substrates and organize the initial ubiquitination of specific substrate regions. ERAD E2s, which serve as ubiquitin-conjugating enzymes, are responsible for generating select topologies of ubiquitin chains on substrates. Among them, UBE2J1 and UBE2J2 are membrane-embedded E2s that attach ubiquitin to lysine or to serine, threonine, or cysteine residues in substrates lacking lysine residues.^86,131–133^ UBE2G2 is recruited to the ER, where it associates with ERAD E3 ubiquitin ligases and facilitates efficient ubiquitin transfer.^134–137^ UBE2K attaches ubiquitin to the Lys48 position of Lys63-linked polyubiquitin chains to form branched polyubiquitin chains, which potentially serve as more efficient signals to transport substrates to the 26S proteasome.^85,100,138^

#### Substrate presentation to the ubiquitination machinery

Disordered regions within ERAD E3 ubiquitin ligases function in substrate translocation and binding. Examples include HRD1 and its cofactors, including family 8 member A1 (FAM8A1), homocysteine-responsive endoplasmic reticulum-resident ubiquitin-like domain member 1 (HERPUD1, alternatively termed HERP), and UBE2J1. These proteins possess intrinsically disordered stretches between the TMD and an intact folded functional domain, allowing flexible binding to an emerging substrate while modifying the substrate conformation to allow efficient ubiquitination.^139^ The position of the RING domain within an ERAD E3 ubiquitin ligase constrains its ability to move, restricting the range of substrates that can be engaged.^140^ After leaving the ER, substrates should remain soluble, with ubiquitin acceptor amino acid residues accessible to the ubiquitination machinery. Newly exposed hydrophobic transmembrane domains of membrane substrates are prone to aggregation in the cytoplasm, which is prevented by BCL-2-associated athanogene 6 (BAG6).^141,142^ The holdase complex, consisting of BAG6, transmembrane domain recognition complex 35 (TRC35), and ubiquitin-like protein 4 A (Ubl4A), shields hydrophobic regions from a hydrophilic environment and prevents the formation of nondegradable protein aggregates. The holdase complex also cooperates with gp78 and HRD1 for ubiquitination following substrate translocation.^122,143^

#### ERAD E3 ubiquitin ligases

ERAD E3 ubiquitin ligases define the specificity required to eliminate undesirable or cytotoxic proteins, while maintaining their properly folded counterparts intact. Diverse ERAD strategies have evolved to fine-tune protein quality and quantity control. These strategies include ERAD E3 ubiquitin ligases that polyubiquitinate substrates by cooperating with E3 ubiquitin ligases that catalyze monoubiquitination or with E4 ligases that play a role in highly processive polyubiquitin chain elongation but not in the initial steps of ubiquitination,^144,145^ often requiring sequential rounds of ubiquitination and deubiquitination.^122,146,147^ Overall, different E3 ubiquitin ligases function in ERAD processing, from protein translocation to protein degradation, exemplified by the selective association of the ATPase p97/VCP with E3 ubiquitin ligases.

HRD1, known as synoviolin (SYVN1), exemplifies the most conserved branch of mammalian ERAD (Fig. 4). Although it contains relatively few lysine residues in its cytosolic domains, HRD1 autoubiquitination has similar characteristics to those of other ubiquitin ligases, which are required for its function.^148,149^ This autoubiquitination is controlled by the ER-anchored ubiquitin-specific protease 19 (USP19), which protects HRD1 from proteasomal degradation.^150^ The ability of HRD1 to escort proteins from the ER requires SEL1L interaction, which stabilizes HRD1 and neutralizes its autoubiquitination.^67,151–154^ SEL1L interacts with a number of proteins, including ancient ubiquitous protein 1 (AUP1), DERL1, DERL2, VCP-interacting membrane protein (VIMP), and UBXD8, which are implicated in p97/VCP recruitment.^81,121,135,155,156^ SEL1L interactions with chaperones enable its recognition of substrates within the ER. Overall, the selectivity of the SEL1L-HRD1 complex for selecting substrates defines important biological processes. For example, HRD1-mediated ERAD of pre-B-cell receptor (BCR) is essential for B-cell development and determines pre-BCR abundance, attenuating pre-BCR-driven signaling during the transition from the large to small pre-B-cell stage.^157^ HRD1 also eliminates MHC-I heavy chains that do not attain the proper conformation once in a complex with β2-microglobulin, indicating its role in controlling antigen presentation.^90,133^ HRD1 involvement in immunological processes is exemplified by HRD1-mediated ubiquitination of a serine residue on the T-cell receptor α (TCRα)^158^ and of serine, threonine, and lysine residues on the immunoglobulin Ig light chain (NS1LC).^86^ Key cellular components involved in DNA damage, the cell cycle, and detoxification programs are also controlled by HRD1 via its regulation of p53, BLIMP1, Nrf2, and PGC1β.^105,106,159–161^

Another major E3 ubiquitin ligase that serves a critical function in ERAD is gp78, referred to as RNF45 or autocrine motility factor receptor (AMFR).^162^ Despite its high similarity with HRD1, gp78 possesses a unique repertoire of membrane partners. The gp78 RING domain resides within its C-terminal cytoplasmic tail, and its five TMDs are located at the N-terminus.^163^ Gp78 interacts with UBE2G2 via its UBE2G2 binding region (G2BR), resulting in conformational changes within UBE2G2 that increase its interaction with gp78.^164,165^ Gp78 mediates the ERAD of misfolded proteins, including ataxin-3, superoxide dismutase-1 (SOD1), and mutant huntingtin protein, and is implicated in several chronic diseases.^166^ Gp78 binds to the C-terminal region of the nonglycosylated prion protein PrP, which is implicated in bovine spongiform encephalopathy and Creutzfeltd–Jacob disease, promoting PrP degradation.^167^ Gp78-mediated ubiquitination of Ubl4A results in irreversible proteolytic processing and inactivation of BAG6.^168^ The control of this activity is regulated by USP13, which associates with gp78 and deubiquitinates Ubl4A, a process that optimizes PQC. Viral or microbial DNA facilitates gp78 interaction with INSIG-1 and stimulator of interferon genes (STING), resulting in K27-linked polyubiquitination of STING, a noncanonical ubiquitin chain topology of ubiquitin that serves as an anchoring platform to recruit TANK-binding kinase 1 (TBK1) and facilitate its translocation to perinuclear microsomes, resulting in the innate immune response.^169^ Gp78 also interacts with RNF5, which facilitates the degradation of virus-induced signaling adaptor (VISA, referred to as mitochondrial antiviral signaling proteins (MAVS)).^170^ Gp78 cooperation with RNF5 has also been implicated in the ERAD of mutant cystic fibrosis transmembrane conductance regulator (F508del CFTR), which is a commonly mutated form of CFTR in cystic fibrosis patients.^146^

MARCHF6, known as TEB4 or RNF176, is a large ER-resident ERAD E3 ubiquitin ligase with multiple transmembrane domains and comprises an N-terminal RING domain and C-terminal sequences that function in self-regulation.^171,172^ USP19 and cholesterol limit the degree of MARCHF6 autoubiquitination, increasing its stability.^173,174^ MARCHF6 reportedly facilitates degradation of the HRD1 complex component FAM8A1, suggesting a concerted action among ERAD E3 ubiquitin ligases.^139^ In cooperation with p97/VCP, MARCHF6 also promotes the translocation of tail-anchored proteins into the cytoplasm.^95–97^ The E3 ubiquitin ligase activity of MARCHF6 is controlled by its interaction with NADPH. Among the MARCHF6 substrates are factors involved in ferroptosis, including acyl-CoA synthetase long chain family member 4 (ACSL4) and p53. MARCHF6 degradation of cytosolic proopiomelanocortin (POMC, a precursor of adrenocorticotropic hormone (ACTH)), melanocyte-stimulating hormone (MSH), and endorphin (END) has been implicated in metabolic homeostasis in neurons.^175^

RNF5, known as RING membrane-anchor 1 (RMA1), is a 22 kDa membrane-anchored ERAD E3 ubiquitin ligase. RNF5 comprises an N-terminal RING domain and C-terminal TMDs and is regulated by autoubiquitination.^170^ Misfolded proteins targeted by RNF5 include mutant CFTR,^119^ solute carrier family 1 member 5 (SLC1A5), and SLC38A2.^176^ RNF5 also controls the stability of sterol regulatory element-binding protein (SREBP)-cleavage activating protein (SCAP),^177,178^ JNK-associated membrane protein (JAMP),^179^ MAVS, an activity that affects the antiviral response,^180^ and STING, which affects cardiac hypertrophy.^181^ RNF5 acts in concert with other ubiquitin ligases to ensure optimal ERAD. Gp78 was suggested to serve as an E4 for RNF5.^146^ RNF5 and the cytoplasmic E3 ubiquitin ligase HECT and RLD domain-containing E3 ubiquitin ligase 3 (HERC3) cooperate to facilitate the ERAD of F508del CFTR.^182^ Additionally, combined inactivation of RNF5 and RNF185 leads to the stabilization of F508del CFTR, suggesting that RNF5 and RNF185 cooperate in the regulation of select substrates, as exemplified by F508del CFTR.^99,183^ RNF5 was suggested to embed its TMD not only in the ER but also in the nucleus,^184^ suggesting that PQC machinery components in the ER may transition between different subcellular compartments for organellar homeostasis. RNF5 has also been shown to be engaged in cellular signaling by regulating the stability of apoptosis signal-regulating kinase 1 (ASK1), which protects against acute myocardial infarction.^185^

Other ERAD E3 ubiquitin ligases have been implicated in sterol biosynthesis, with implications for immune regulatory functions. TRC8, known as RNF139, is a large multimembrane-spanning protein containing an N-terminal sterol-sensing domain (SSD) and a C-terminal RING finger motif.^186^ SSD adjusts TRC8 levels following changes in cholesterol concentration, although interconnections between TRC8 and cholesterol homeostasis remain elusive.^187^ TRC8 and SPP also participate in the formation of a complex with DERL1, which extracts tail-anchored proteins from the ER.^95,96,124^ The TRC8 complex is hijacked by cytomegalovirus (CMV) to target MHC-I for degradation as an immune evasion strategy.^42,188^ The CMV-encoded US2 protein functions as an adaptor between MHC-I and UBE2G2-TRC8, allowing the targeting of MHC-I for ERAD.^189^

ERAD’s ability to impact the immune system is exemplified by TMEM129, a polytopic membrane protein with three N-terminal TMDs followed by a noncanonical RING domain with intrinsic E3 ubiquitin ligase activity.^190^ Cooperation of TMEM129 with DERL1 and UBE2J2 constitutes the rate-limiting step of MHC-I degradation.^120^ US11, the gene product of human CMV, recruits DERL1, TMEM129, and UbE2J2 to engage the neonatal Fc receptor (FcRn), which results in the initiation of FcRn dislocation from the ER to the cytoplasm, leading to its degradation.^191^ Moreover, US11 attenuates immunoglobulin G (IgG)-FcRn binding, thereby leading to a reduction in IgG transcytosis across intestinal or placental epithelial cells and the degradation of IgG in endothelial cells. TMEM129 can therefore be a major player in MHC-I regulation and function.

### Substrate unfolding and extraction from the ER to the cytoplasm

P97/VCP is among the most functionally diverse eukaryotic AAA+-ATPases implicated in protein extraction from diverse membranes, including the ER, nuclear, and mitochondrial membranes.^192^ It interacts with a wide variety of adaptor proteins and associated enzymes that facilitate its activities at diverse nuclear and cytoplasmic sites.^192^ AAA+-ATPases assemble into hexameric ring-shaped complexes consisting of a central pore and can translate chemical energy into mechanical force, enabling protein unfolding and extraction through complex membranes.^193^ Each p97/VCP subunit possesses an N domain for cofactor binding; two ATPase domains, each with a D1 domain, which, are required to regulate the adaptor configuration and to coordinate ATPase activity; and a D2 domain, which is required to maintain the energy supply needed to unfold proteins.^194^

The P97/VCP N domain interacts with several E3 ubiquitin ligases via dedicated binding motifs. These include the VIM within HRD1, the VCP-binding motif (VBM) within gp78, SHP within DERL1/2, and UBX within UBXD8.^195^ NPL4–ubiquitin fusion degradation 1 (UFD1) heterodimers interact with the p97/VCP N domain and capture ERAD substrates via their Lys48-linked polyubiquitin signature.^196^ This interaction promotes the unfolding of ubiquitin within the polyubiquitin chain, as the ubiquitin is inserted into a groove in NPL4–UFD1.^197,198^ Interestingly, tripartite motif containing 21 (TRIM21) was shown to facilitate Lys27-linked UFD1 polyubiquitination and inhibit the interaction of UFD1 with p97/VCP, thus suppressing ERAD and activating the proapoptotic UPR.^199^ The ubiquitin moiety unfolded by NPL4–UFD1 then enters the p97/VCP central pore, followed by ATP hydrolysis by D2 domains, which pulls ubiquitinated substrates through the narrow cavity, promoting their unfolding.^198,200^ YOD1 binds to the N domain and cleaves Lys48-linked ubiquitin on substrates, contributing to substrate release from NPL4–UFD1 and their entry into p97/VCP.^201^ ATPase cycles coordinated by p97/VCP result in effective translocation of oligo-ubiquitinated substrates to the opposite side of the ring. In parallel, p97/VCP increases the solubility of membrane substrates in the aqueous cytoplasmic environment following translocation.^202^ *N*-linked oligosaccharides on ERAD substrates presumably hinder substrate entry into the p97/VCP central pore. The cytosolic deaminating enzyme peptide *N*-glycanase (NGly1) is recruited to p97/VCP, ensuring the removal of *N*-linked oligosaccharides after their translocation to the cytoplasm prior to engagement by p97/VCP.^203,204^

### Delivery of substrates to the 26S proteasome for degradation

To avoid the deleterious accumulation of unfolded intermediates, the protein extracted from the ER needs to enter the 26S proteasome to enable effective degradation. Ubiquitin-like (UBL) and ubiquitin-associated (UBA) domains within BAG6 and RAD23 prevent refolding or aggregation of emerging ERAD substrates, facilitating proteasomal delivery.^205^ Extracted substrates rapidly engage E3 ubiquitin ligases, such as RNF126, which results in their ubiquitination, which targets them for proteasomal degradation.^143,206^ Several cytosolic E3 ubiquitin ligases can generate branched or mixed ubiquitin chains, promoting substrate access to the 26S proteasome.^85,100^ RPN1, RPN10, and RPN13 function as receptors that position ERAD substrates for entry into the proteasomal 19S regulatory particle.^35^ Ubiquitin C-terminal hydrolase L5 (UCHL5) and USP14 might proofread ubiquitinated proteins, resulting in the removal of ubiquitin moieties to block their degradation, whereas RPN11 might eliminate ubiquitin moieties from ubiquitinated substrates before proteasomal degradation.^13,207,208^ The cytosolic protein degradation machinery clusters within ∼200-nm foci that interact with particular ER membranes.^209^ These microcompartments consist of densely clustered proteasomes and substrates, suggesting that proteasomes are localized at select ER hot spots to enable efficient ERAD.

## Erad as part of the unfolded protein response (UPR)

Proteotoxic ER stress induces the UPR, which coordinates stress responses to maintain ER function.^45,210^ The UPR engages one or more of the three principal sensors embedded in the ER membrane, including activating transcription factor 6 (ATF6), inositol-requiring protein 1 (IRE1) α and β, and protein kinase RNA (PKR)-like ER kinase (PERK).^211^ ERAD and the UPR are intimately linked, enabling ERAD to ensure protein quality and quantity, to induce gene expression programs, and to maintain normal physiology.^212–214^ ATF6 activation requires its cleavage to enable transcriptional control of ER quality control proteins.^215–217^ IRE1 induces *XBP1* splicing, resulting in spliced XBP1 (XBP1s), which regulates the expression of chaperones, including ERdj3, ERdj4, EDEM, PDI, and ribosome-associated membrane protein 4 (RAMP-4).^218^ ATF6/XBP1 heterodimerization increases the levels of major ERAD components under conditions of proteotoxic ER stress.^219^ Posttranscriptionally, sustained ER stress increases RNF183 protein levels in an IRE1α-dependent manner and the degradation of Bcl-xL.^220^ While IRE1 and ATF6 upregulate HRD1, prolonged ER stress downregulates bifunctional apoptosis regulator (BAR), which is known to mediate proteasomal degradation of the ER-resident IRE1 inhibitor protein Bax inhibitor-1 (BI-1), which controls IRE1 signaling.^221^ PERK, the most studied UPR sensor, activates ATF4, resulting in widespread transcriptional activation of genes engaged in diverse pathways, including metabolism, autophagy, and cell death programs.^222^

ERAD crosstalk with the UPR results in the concerted action of diverse ERAD components. Among those is SPP, which forms a complex with DERL1 and TRC8 to cleave XBP1u, a translation product of unspliced *XBP1* RNA that negatively regulates the UPR.^124^ Another example is the ER-resident E3 ubiquitin ligase RNF13, which interacts with IRE1α to enable TRAF2 signaling.^223^ Notably, the interface between the ERAD and UPR also includes a negative feedback loop, exemplified by limiting IRE1α and ATF6 signaling upon their targeting for ERAD by HRD1.^224,225^ Under basal physiological conditions, HRD1-mediated IRE1α degradation occurs constitutively to restrain IRE1α activity.^224^ Restraining the magnitude and persistence of basal IRE1α activity is required to maintain cellular homeostasis, as aberrant IRE1α activation has deleterious outcomes.^226^ ER stress promotes the release of BiP and the HRD1 complex from IRE1α, which results in IRE1α stabilization. Constitutive IRE1α regulation by HRD1-mediated ERAD was shown to protect the gut epithelium from inflammatory diseases.^227^ Similarly, HRD1 suppresses IRE1α-mediated ER stress responses by facilitating the degradation of unfolded proteins in Treg cells, maintaining Treg stability and function, suggesting that HRD1 controls the IRE1α pathway.^228^ Along these lines, disruption of ERAD via depletion of Sel1L or HRD1 leads to the activation of PERK and drives hematopoietic stem cell (HSC) proliferation.^229^ Mechanistically, PERK activation via ERAD deficiency promotes aberrant mammalian target of rapamycin (mTOR, alternatively termed the mechanistic target of rapamycin) signaling and HSC hyperproliferation. These factors compromise self-renewal capacity, indicating the role of PERK in modulating HSC fate under conditions of ER stress. It is essential to establish whether the interplay of ERAD with the UPR is linked to disease initiation and progression, as their dysfunction has been independently reported in human diseases.

In addition to its role in UPR-driven transcriptional changes via *XBP1* splicing, IRE1α is involved in the regulated IRE1α-dependent mRNA decay (RIDD) to halt further translation, thereby inhibiting excessive protein burden in the ER and ensuring proteostasis under conditions of ER stress.^230–233^ RIDD provides more than passive ER stress relief by forestalling protein synthesis in the ER, as the activity of RIDD is increased in proportion to the duration and severity of ER stress.^234^ IRE1α dimers have the ability to cleave specific RIDD targets that contain stem–loop endomotif.^226,235^ Interestingly, oligomerization of IRE1α plays an important role in its activity. First, phosphorylated IRE1α oligomers display greater RNase cleavage capacity than dimers do.^236^ Higher-order oligomerization of phosphorylated IRE1α can cleave mRNAs without the canonical stem–loop sites through a process referred to as RIDDLE (RIDD lacking endomotif), suggesting two RNase modes: specific endomotif-dependent cleavage and promiscuous, endomotif-independent cleavage. However, the mechanisms underlying target specificity and transcript recruitment for RIDD and RIDDLE have not been fully characterized, although RIDDLE is also capable of processing cleaved RIDD substrates.^236^ Although emerging evidence suggests the important roles of RIDD and RIDDLE in physiology and pathology, few mRNA targets for RIDD and RIDDLE have been identified and characterized at the ER. RIDD enhances cellular degradative capacity by cleaving *BLOS1* mRNA,^237,238^ suppresses lipid synthesis by cleaving *DGAT2* mRNA,^239^ regulates cell fates by degrading *DR5* mRNA,^240^ and attenuates antigen cross-presentation by cleaving *MHC-I heavy chain* mRNA.^241^ As RIDD has emerged as a potent regulator of ER PQC, determining the integration of RIDD with ERAD while elucidating the broader physiological and pathological implications of their interplay is important.

## Erad interplay with ER-phagy

**Fig. 5:**
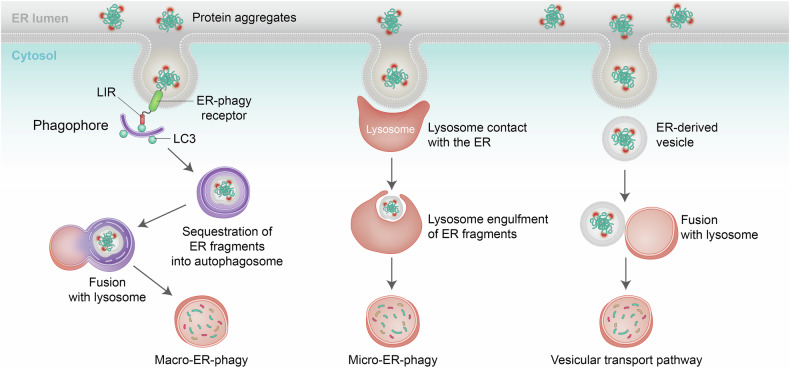
ER-phagy. During macro-ER-phagy, ER-phagy receptors associate with LC3 and pack ER fragments into a phagophore. The phagophore elongates and subsequently seals, forming double-membrane vesicles, known as autophagosomes, that transport ER fragments to the lysosome for breakdown. In the process of micro-ER-phagy, the lysosomal membrane comes into direct contact with the ER and ER fragments. The lysosome then directly engulfs the ER fragments. During the vesicular transport pathway, ER-derived vesicles directly fuse with lysosomes, giving rise to a specialized form of ER-phagy

Along with ERAD-mediated clearance of misfolded, unfolded, or incompletely assembled proteins, there is increasing recognition that, via lysosomal degradation, selective autophagy of the ER (ER-phagy) also serves to dispose of misfolded proteins or protein aggregates unrecognizable by the ERAD or too large to undergo translocation from the ER into the cytoplasm for ERAD.^9,12,14–16^ ER-phagy is also capable of extracting damaged ER membranes and facilitating ER shrinkage following the resolution of ER stress, thereby maintaining the homeostasis of different ER membranes. Three distinct forms of ER-phagy have been identified: macro-ER-phagy, micro-ER-phagy, and the vesicular transport pathway (Fig. 5). In macro-ER-phagy, ER fragments are sequestered into autophagosomes, which are double-membrane vesicular structures.^11,12,242–244^ The fusion of the autophagosome’s outer membrane with the lysosomal membrane leads to the degradation of the inner autophagosomal membrane as well as sequestered ER fragments. During micro-ER-phagy, ER fragments are directly engulfed by lysosomes or endosomes.^11,12,242^ The vesicular transport pathway involves the direct fusion of vesicles derived from the ER with lysosomes, constituting a unique form of ER-phagy.^245^ Accordingly, one would expect that ER-phagy functions as a critical arm of ER quality control and proteostasis,^12,246,247^ and therefore, in human diseases. In addition, mutations in genes encoding ER-phagy components have been associated with various human diseases, including diabetes and neurological disorders.^9,248^

The execution of ER-phagy requires multiple downstream components that are shared with conventional autophagic pathways, as exemplified by microtubule-associated protein 1A/1B-light chain 3 (LC3) and membrane sources for the delivery of cargos to lysosomes. In addition, ER-phagy is dependent not only on receptor proteins with the capacity to alter ER membrane morphology through bending or distortion but also on proteins with the capacity to mediate membrane scission. Mammalian ER-phagy receptors involve eight transmembrane proteins, including reticulophagy regulators 1, 2, and 3 (FAM134B/RETREG1, FAM134A/RETREG2, and FAM134C/RETREG3), SEC62, the long isoform of reticulon 3 (RTN3L), cell cycle progression 1 protein (CCPG1), atlastin GTPase 3 (ATL3), and testis expressed 264 (TEX264), as well as three soluble proteins, including sequestosome 1 (p62/SQSTM1), calcium binding and coiled-coil domain (CALCOCO)1, and CALCOCO3.^249–256^ The soluble receptor proteins are linked to the ER through specific ER-resident proteins such as vesicle-associated membrane protein-associated protein A/B (VAPA/VAPB), TRIM13, or the ubiquitin-fold modifier 1 (UFM1)-DDRGK domain containing 1 (DDRGK1) complex. Common to all ER-phagy receptors, they possess an LC3-interacting region (LIR), which permits the ER-phagy receptors to bridge ER fragments with autophagosomes.^242^

Among the ER-phagy receptors, FAM134B is the most studied. FAM134B is confined in topology to the outer leaflet of the ER membrane and possesses a reticulon homology domain (RHD) that supports the positioning of FAM134B at the curved edges of the ER sheets and LIRs within the cytosolic C-terminal region.^250^ FAM134B is oligomerized, which enables FAM134B to function as an ER-phagy receptor.^257–259^ FAM134B plays a crucial role in the selective autophagic clearance of a wide range of misfolded proteins, such as procollagen and its mutants,^260^ mutant Niemann–Pick disease type C intracellular cholesterol transporter 1 (NPC1),^261^ and mutant alpha-1-antitrypsin variant Z (Z-AAT).^245^ Interestingly, FAM134B physically associates with calnexin, thereby bridging FAM134B with misfolded procollagens.^260^ FAM134B has been demonstrated to function in the constitutive targeting of ATZ polymers to the lysosome, indicating its role in the maintenance of ER function.^245^ Importantly, mice lacking FAM134B exhibit sensory neuron loss at 12–15 months of age, indicating an age-related neurological phenotype.^250^ Inactivation of both FAM134B and FAM134C also results in severe growth defects, early-onset neuromuscular and somatosensory degeneration, and premature death at an age younger than 25 weeks, indicating their cooperation and importance.^262^ Genetic alterations in *FAM134B* are associated with hereditary sensory and autonomic neuropathy type II (HSAN-II), thereby leading to severe and progressive impairments in sensations of pain, temperature, and touch in the distal limbs.^263,264^

Another ER-phagy receptor is RTN3. Like FAM134B, RTN3 is topologically confined within the outer ER leaflet and possesses RHD; however, RTN3 is localized primarily at the ER tubules, unlike the ER sheets, where FAM134B is localized. RTN3 targets misfolded proteins, including mutant proinsulin, mutant POMC, and pro-arginine vasopressin (proAVP), for lysosomal degradation.^248^ To accomplish the selective recruitment of mutant proinsulin and POMC, RTN3 associates with the ER membrane protein progesterone receptor membrane component 1 (PGRMC1). PGRMC1 directly associates with improperly folded proteins in the ER lumen, bridging them with RTN3.^265^ RTN3 is highly enriched in neurons and is associated with Alzheimer’s disease (AD).^266^

A different ER-phagy receptor is ATL3, a transmembrane protein with intrinsic GTPase activity. ATL3 was initially linked to ER tubules^267^ and was later shown to play an important role in their availability.^253^ Like RTN3 and FAM134B, ATL3 harbors a double hairpin structure resembling a partial RHD, which facilitates the localization of ATL3 to the ER tubules and enables its interaction with LC3. Two variants of *ATL3* have been reported in patients with HSAN-I.^268,269^ The importance of ATL3 is revealed through *ATL3* mutants, which cause morphological alterations in the ER, ultimately resulting in ER tubular network collapse.^270,271^

Sec62, which is traditionally identified as part of the ER translocon complex, is another ER-phagy receptor that contributes to ER return to its normal size subsequent to the relief of ER stress. Sec62 is considered orphaned under conditions of ER stress.^251^ When ER stress is alleviated, orphaned Sec62 connects the ER to LC3 and the lysosome, thereby promoting ER-phagy. This process is termed recovER-phagy, in which ER fragments harboring orphaned Sec62 are engulfed by the lysosome *via* micro-ER-phagy.

Among the ER-phagy receptor proteins, TEX264 plays a predominant role in mediating the autophagic degradation of the ER under both basal and nutrient starvation conditions.^272^ TEX264 possesses an LIR-containing C-terminal region exposed to the cytoplasm and a long intrinsically disordered region (IDR), which contributes to the passing of the LIR motif through highly dense ribosomes bound to the ER and extending to LC3 on the isolation membrane (phagophore).^254,272^ However, further studies are needed to clarify the physiological relevance of TEX264-dependent ER-phagy.

CCPG1 is the sole identified ER-phagy receptor capable of directly interacting with ER luminal cargo, as CCPG1 possesses an extensive ER luminal region enriched with multiple conserved regions for cargo binding.^273^ Cargos for CCPG1 include ectopically expressed islet amyloid polypeptide and endogenous prolyl 3-hydroxylase family member 4 (P3H4).^273^ In addition, CCPG1 plays a critical role in regulating ER mass under conditions of ER stress, and its deficiency in mice results in disrupted pancreatic ER proteostasis.^252^

The interplay between ERAD and ER-phagy has been suggested, although the occurrence and physiological implications of their interplay remain to be fully elucidated. Several disease-associated mutant proteins are subject to regulation via both ERAD and ER-phagy, including Akita proinsulin in diabetes,^248,265,274,275^ POMC C28F in early-onset obesity, proAVP G57S in diabetes insipidus,^276,277^ and NPC1 I1061T in Niemann‒Pick type C disease.^261^

Low-molecular-weight oligomers of Akita proinsulin are eliminated from the ER by ERAD, whereas its larger high-molecular-weight oligomers undergo exclusive degradation via RTN3-mediated ER-phagy.^248^ Notably, ER-phagy becomes activated to degrade high-molecular-weight proinsulin aggregates in ERAD-deficient β cells.^278^ Deficiencies in both ERAD and ER-phagy aggravate proinsulin aggregation within the ER and disrupt insulin secretion, ultimately resulting in progressive β-cell death.

ER-phagy and ERAD also collaborate to maintain proAVP maturation and to protect AVP neurons against proteotoxicity, thereby leading to an increase in AVP neuron survival.^279^ Mechanistically, FAM134B-mediated ER-phagy selectively degrades mutant proAVP aggregates. However, HRD1 upregulation is also associated with a reduction in proAVP aggregation in the context of autophagy deficiency, thereby maintaining AVP neuron function and survival.

Procollagen is targeted for degradation by ERAD; however, certain forms of this protein are resistant to ERAD and are eliminated through ER-phagy.^280^ A substantial portion of procollagen fails to fold properly and is subsequently eliminated through ER-phagy to suppress procollagen accumulation within the ER. Macro-ER-phagy and micro-ER-phagy were shown to degrade misfolded, mutant procollagen, although it is unclear how and why the mutant procollagen undergoes degradation via both macro-ER-phagy and micro-ER-phagy.^260,281^

ERAD and ER-phagy work in concert to control misfolded lipoprotein lipase (LPL) and its aggregates within the ER.^282^ ERAD deficiency in adipocytes leads to the accumulation and aggregation of misfolded LPLs within the ER, which are removed via ER-phagy.

Both ERAD and ER-phagy are crucial for the clearance of the Z variant of alpha-1 antitrypsin (AAT), a protein primarily produced in the liver (Z-AAT). The Z variant is a mutated form of AAT that causes misfolding and aggregation of the protein within liver cells, resulting in liver damage and fibrosis. The application of an autophagy inducer to Z-AAT transgenic mice enhances autophagic flux and decreases the number of Z-AAT aggregates, thereby attenuating liver fibrosis and inflammation.^283–285^ Upregulation of HRD1 results in Z-AAT degradation.^286^

Overall, these examples highlight the interplay of ERAD with ER-phagy in maintaining the balance of the ER proteome and mitigating aggregate-associated toxicity.

## Physiological implications of erad

Mechanisms to maintain proteostasis are essential for aligning cellular demands with environmental challenges and mitigating stress conditions. Importantly, proteins directed to the ER, where the synthesis, folding, and assembly of secretory and transmembrane proteins take place, play essential roles in cellular homeostasis and signaling, which are closely associated with nearly all aspects of cell physiology. Proteins that are terminally misfolded or incorrectly targeted in the ER constitute a major risk to cells and therefore need to be eliminated.^36,287,288^ Thus, the quality and quantity of the proteome at the ER must be adequately maintained by ER PQCs, including the UPR, ERAD, and ER-phagy, ensuring unperturbed homeostasis. Studies of ERAD indicate that it maintains the integrity and functionality of ER-resident or cytoplasmic proteins in response to various stimuli under both physiological and pathophysiological conditions.^8,289^ By ensuring protein quality and quantity, ERAD exerts posttranslational control over lipid homeostasis, interorganelle contacts, calcium flux, and nuclear envelope organization, which affect nearly all intrinsic cellular functions.^289^

### Linking ERAD to calcium homeostasis

High ER Ca^2+^ levels are required for proper ER function, and disruption of Ca^2+^ homeostasis promotes the deposition of misfolded proteins within the ER. The active transport of Ca^2+^ into the ER is carried out by sarco- and endoplasmic Ca^2+^ATPase (SERCA).^290^ Notably, a pronounced Ca^2+^ gradient across the ER membrane drives the efflux of Ca^2+^ from the ER into the cytoplasm, leading to the generation of Ca^2+^ signals that extend throughout the cell. The efflux of Ca^2+^ from the ER is mediated by IP_3_Rs,^291,292^ ryanodine receptors (RyRs),^292,293^ and/or members of a large group of Ca^2+^-leak channels.^294^ A distinct characteristic of intracellular Ca^2+^ signaling is the ability of the ER to regulate the flux of Ca^2+^ at mitochondria-associated ER membranes (MAMs), the contact sites between the mitochondrial outer membrane and the ER. The major requirement for the regulation of Ca^2+^ communication at MAMs is the physical association between a tetrameric ion channel, IP_3_R, and voltage-dependent anion channels (VDACs), which is reinforced and connected by glucose-regulated protein 75 (GRP75), a member of the HSP70 family.^295^ Moreover, various PQC components of the ER contribute to Ca^2+^ signaling and homeostasis.^296^ PERK deficiency leads to the development of multiple alterations in the morphology and number of MAMs, with disrupted Ca^2+^ transfer between the ER and mitochondria.^297^ PERK signaling also upregulates the expression of a truncated isoform of ATPase sarcoplasmic reticulum/ER Ca^2+^ transporting 1 (S1T) and Sigma-1 receptor (Sig-1R), which serve to ensure Ca^2+^ transfer.^298^ IRE1 is strongly enriched at MAMs and serves as a scaffold, docking IP_3_R1 and IP_3_R3 at MAMs, serving as a determinant for the amplitude of Ca^2+^ transfer.^299^ Moreover, IRE1 prevents mitochondrial Ca^2+^ overload in neuronal cells.^300^ Notably, the presence of UPR sensors at MAMs and their regulatory roles in Ca^2+^ transfer might open a new paradigm of ER‒mitochondria communication during proteotoxic stress.

ERdj5, a PDI with important roles in ERAD, reduces the number of luminal disulfide bonds in SERCA2b, the ubiquitous isoform of SERCA, and facilitates the pump function of SERCA2b.^301^ Notably, ERdj5 activates SERCA2b at a lower level of Ca^2+^ within the ER lumen, whereas a higher level of Ca^2+^ within the ER lumen results in the formation of ERdj5 oligomers that are no longer capable of interacting with SERCA2b, suggesting the importance of crosstalk among Ca^2+^ levels and proteostasis in the ER. Sig-1R, the major determinant of MAM structure, is degraded by ERAD.^302^ Interestingly, Sig-1R stabilizes IRE1 and enhances the endonuclease activity required for the UPR, which, in turn, prevents PERK signaling and regulates Ca^2+^ signaling.^303^ IP_3_R, which is required for Ca^2+^ release from the ER lumen upon IP_3_ activation, is a substrate for RNF170.^140^ Conformational changes associated with IP_3_R activation lead to its recognition by the RNF170/ERLIN complex, promoting IP_3_R degradation and decreasing Ca^2+^ release. Attenuation of the E1 ubiquitin-activating enzyme UBE1 blocks IP_3_R1 ubiquitination and degradation without disrupting the ERLIN1/2 complex association, indicating that IP_3_R1 processing is initiated primarily by its association with the ERLIN1/2 complex. Mutations in RNF170, ERLINs, or IP_3_R are implicated in neurological disorders, including ataxias and spastic paraplegias.^304–306^ Overall, these studies demonstrate the interconnections between ERAD and calcium homeostasis.

### Linking ERAD to lipid homeostasis: bidirectional communication between proteotoxicity and lipotoxicity

ERAD functions as a posttranslational feedback regulator of lipid homeostasis during metabolic and environmental fluctuations. Lipotoxicity is defined as dysregulation of the lipid environment or lipid composition that promotes the accumulation of toxic lipids. Lipotoxicity can trigger proteotoxic stress by altering ER integrity, fluidity, and/or structure, regardless of the levels of adequately folded ER proteins,^307–309^ suggesting that there is crosstalk between proteotoxicity and lipotoxicity.

Sterols are essential membrane components that modulate membrane biophysical properties, including fluidity, rigidity, and permeability. Moreover, free sterol accumulation induces lipotoxicity, suggesting that sterol abundance must be tightly regulated.^310,311^ HMGCR is an ER membrane–anchored protein responsible for catalyzing the rate-limiting step of sterol biosynthesis, namely, the reduction of β-hydroxy β-methylglutaryl-CoA (HMG-CoA) to mevalonate, a key intermediate in the synthesis of cholesterol and nonsterol isoprenoids, such as geranylgeranyl pyrophosphate (GGpp).^312^ ERAD regulation of sterol biosynthesis occurs when flux through the mevalonate pathway is high.^312,313^ Sterol accumulation facilitates HMGCR binding to ER membrane-embedded INSIG-1/2, promoting the association of HMGCR with gp78 and RNF145 and subsequent ERAD.^314,315^ INSIG itself is a target for ERAD via gp78, revealing an additional layer of HMGCR regulation.^187^ Sterols stimulate the binding of UbiA prenyltransferase domain-containing protein 1 (UBIAD1) to HMGCR and attenuate HMGCR degradation.^316^ The accumulation of geranylgeraniol, the alcohol derivative of GGpp, inhibits the binding of UBIAD1 to HMGCR, restoring HMGCR degradation. MARCHF6 also functions in lipid metabolism.^171,317–319^ MARCHF6 and Ube2J2 facilitate the ERAD of squalene epoxidase (SQLE) under conditions where further cholesterol synthesis is not needed.^320^ The N-terminal degron within the SQLE allows recognition by MARCHF6.^98,320,321^ Perilipin-2 (PLIN2), mutant NPC1, and bile salt export pump (BSEP) are substrates for MARCHF6.^261,322,323^ MARCHF6 and the recognition component of the Ac/N-end rule pathway directly target N-terminally acetylated PLIN2 for ERAD.^322^ MARCHF6 indirectly regulates liver X receptor (LXR) and SREBP.^324^ Cholesterol and oxysterol promote INSIG binding to a region within the SCAP membrane domain known as SSD.^325^ SSD overexpression attenuates the ERAD of HMGCR accelerated by sterols.^326^ TMEM33, which recruits RNF5 to promote SCAP degradation,^177^ is transcriptionally regulated by an ER-associated transcription factor, nuclear factor erythroid 2-related factor 1 (Nrf1, referred to as NFE2L1), whose cleavage and activation are controlled by pyruvate kinase M2 (PKM2). Insufficient free cholesterol esterification induces ER stress in sterol O-acyltransferase 2 (SOAT2)-depleted enterocytes, leading to RNF5-mediated ubiquitination of CD36, suggesting that RNF5 is associated with lipid uptake.^327^ Other sterol biosynthetic enzymes targeted for ERAD include 7-dehydrocholesterol reductase (DHCR7), DHCR14, and CYP51 A1.^100,328,329^

The feedback loop between ERAD and lipid homeostasis is an emerging area of great interest and has been the subject of a growing number of studies. In addition, cholesterol prevents MARCHF6 autodegradation.^173^ RNF145 accumulates following sterol depletion and is transcriptionally regulated by LXR.^330^ Within unsaturated lipid membranes, RNF145 is stabilized, facilitating its lipid-sensitive binding, ubiquitination, and subsequent degradation of the lipid hydrolase adiponectin receptor 2 (ADIPOR2), indicating that lipid composition regulates RNF145 activity.^331^ Cholesterol also regulates Nrf1 stability, localization, processing, and activity.^332^ Excess cholesterol in the ER reduces Nrf1 translocation to the cytoplasm. In genetic models, liver-specific Nrf1 depletion results in hypersensitivity to cholesterol-induced damage, suggesting that Nrf1 may serve as a guardian of cholesterol homeostasis. Nrf1-deficient liver tissue shows increases in oxidative stress, ER stress, and inflammation, which promote hepatic tumor development.^333,334^ Studies in *Caenorhabditis elegans* have demonstrated that oleic acid (OA) upregulates the Nrf ortholog SKN-1A by enhancing ERAD in a lipid droplet-dependent manner, attenuating steatosis.^335^

### Linking ERAD to organellar homeostasis

Cytosolic PQC by ERAD is performed mainly by the ERAD E3 ubiquitin ligase Doa10.^1,336,337^ Doa10, an ortholog of MARCHF6, is a large transmembrane protein with multiple membrane-spanning domains and is localized at the ER and inner nuclear membrane in yeast.^338^ In addition to its role in ubiquitinating ER proteins, Doa10 also ubiquitinates soluble proteins in the cytosol, given that its substrate recognition occurs within the cytosolic compartment.^337^ Notably, Doa10 is also associated with mitochondrial PQC. Doa10 ubiquitinates certain tail-anchored membrane proteins that undergo initial mistargeting to the mitochondrial outer membrane and are subsequently subject to Msp1 ATPase-mediated extraction from the mitochondrial membrane.^339^ Moreover, certain mitochondrial proteins that fail to be imported into the mitochondrial matrix are targeted for degradation by Doa10 in a process that depends on the presence of an N-terminal mitochondrial-targeting sequence (MTS), similar to other E3 ubiquitin ligases, such as Ubr1 and San1.^340^ Doa10 controls an extensive repertoire of soluble and integral membrane substrates, including cytosolic, mitochondrial, and ER proteins.

Found across all eukaryotic cells, the integrated stress response (ISR) represents a precisely orchestrated signaling network capable of responding to deficiencies in nutrient and oxygen availability, mitochondrial and ER stress, or infection caused by viral pathogens. The ISR maintains or restores the proteostasis of multiple organelles, such as the ER, mitochondria, and Golgi apparatus, thereby controlling cellular behavior, metabolism, and survival.^341–344^ The ISR is distinguished by its ability to regulate transcription and translation, which is implicated in PQC. Irrespective of the stressors that activate the ISR, its activation leads to the phosphorylation of the α-subunit of eukaryotic translation initiation factor 2 (eIF2α) at serine 51, resulting in attenuation of cap-dependent protein translation.^345,346^ Four kinases, including PERK, general control nonderepressible 2 (GCN2), double-stranded RNA-dependent protein kinase (PKR), and heme-regulated eukaryotic translation initiation factor 2α kinase (HRI), are involved in eIF2α phosphorylation, redirecting gene expression to extensively remodel the cellular machinery in support of cellular adaptation.^346^ As discussed earlier, PERK is one of the major stress sensors of the UPR.^211^ ISR activation by PERK results in not only global repression of translation but also transcriptional activation of genes associated with stress response signaling pathways, including the oxidative stress response.^345^ The PERK-eIF2α-ATF4-CCAAT-enhancer-binding proteins (C/EPB) homologous protein (CHOP) axis leads to the transcriptional activation of genes, including ATF4, ATF5 and its related genes; CHOP; growth arrest and DNA damage-inducible protein (GADD34); oligophrenin-1 (OPHN1); inhibitor of Bruton’s tyrosine kinase α (IBTKα); and NUPR1.^347^ Interestingly, PERK does not act in isolation in the ISR but acts in concert with other stress sensors of the UPR, IRE1 and ATF6.^348,349^ Notably, the HRI–eIF2α–HSPB8 axis defines a novel cytosolic UPR that is essential for the correct assembly of certain innate immune signalosomes and for regulating the formation of toxic protein aggregates, which is functionally homologous to the PERK–eIF2α–HSPA5 axis.^350,351^ Furthermore, PERK, an integral MAM component, is essential for preserving ER–mitochondria juxtaposition and for driving mitochondrial apoptosis mediated by ROS.^297^ In response to ROS-induced stress, PERK plays a pivotal role in mitochondrial apoptosis not only by preserving the abundance of proapoptotic CHOP via the ISR but also by promoting the transmission of ROS signals between the ER and mitochondria via its tethering function.

**Fig. 6:**
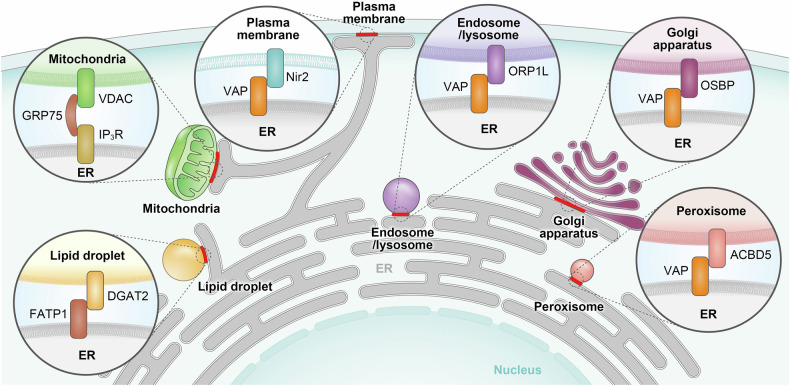
Interorganelle interactions. The diagram depicts the distribution of the ER and membrane contact sites (MCSs) formed by the ER with other organelles and with the plasma membrane. Phosphatidylinositol transfer protein Nir2 (PITPNM1) on the plasma membrane binds to vesicle-associated membrane protein (VAP) on the ER membrane, thereby forming ER–plasma membrane (PM) MCSs. Oxysterol-binding protein (OSBP) localizes to the Golgi apparatus and interacts with VAP on the ER membrane, thereby organizing ER-Golgi apparatus MCSs. Acyl-CoA binding domain-containing protein 5 (ACBD5) on the peroxisomal membrane interacts with VAP on the ER membrane, which results in the formation of ER-peroxisome MCSs. Oxysterol-binding protein-related protein 1 L (ORP1L) on endosomes/lysosomes contacts VAP on the ER membrane to form ER-endosome/lysosome MCSs. ER-mitochondria MCSs (MAMs) are formed by the association of voltage-dependent anion channels (VDACs) on the mitochondria with the inositol 1,4,5-trisphosphate receptor (IP_3_R) on the ER membrane. GRP75 promotes the interaction between VDAC and IP_3_R. Diacylglycerol acyltransferase 2 (DGAT2) on lipid droplets binds to the acyl-CoA synthetase FATP1 on the ER membrane, which results in the formation of ER-lipid droplet (LD) MCSs (see text for details)

Compartmentalization serves as an obstacle for organelle signal transduction and material exchange. To circumvent these obstacles, organelles communicate at membrane contact sites (MCSs), spaced at 10–30 nm.^352,353^ The dynamic formation of MCSs enables the efficient exchange of various signals or factors, including phospholipids, sterols, and calcium. The ER establishes MCSs with other organelles, as with the plasma membrane (Fig. 6), modulating their cytosolic positioning and function.^354,355^ MCSs with the ER undergo elaborate regulation upon various physiological and pathological cues.^356–359^

The MAM connects the ER with mitochondria^360^ and governs mitochondrial dysfunction, autophagy, Ca^2+^ homeostasis, lipid metabolism, inflammatory responses, and apoptosis.^361^ Increased MAM contacts facilitate the initiation and transmission of the ER stress response to reorganize ER and mitochondrial networks. Adipose-specific deletion of HRD1 or SEL1L in mice impairs the degradation of Sig-1R on MAMs, which is implicated in the control of body temperature.^302^ MAM formation is facilitated by the association between IP_3_Rs on the ER membrane and VDACs on the mitochondrial outer membrane.^295^ Ca^2+^ released into the cytoplasm from the ER lumen interacts with VDACs. Upon ER stress, IP_3_R opening induces the active exchange of Ca^2+^ between the ER and mitochondria and contributes to the activation of the NOD-like receptor protein 3 (NLRP3) inflammasome, suggesting that MAMs serve as a mechanistic link bridging NLRP3-induced inflammation and ER stress.^362^ Upon ER stress, IRE1α-XBP1 and ATF6 promote the ubiquitination and degradation of mitofusin 2 (MFN2), a key regulator of mitochondrial fission/fusion control, leading to mitochondrial fission and mitophagy. The latter points to possible crosstalk between the ER and mitochondrial quality control.^363^ Not surprisingly, defects in the folding, translocation, and turnover of mitochondrial proteins pose major challenges to maintaining proteome integrity within mitochondria. Thus, sequestration of damaged proteins from the outer mitochondrial membrane, followed by their targeting to proteasomes for degradation in the cytoplasm, should occur through mitochondria-associated degradation (MAD). Notably, if the targeting and translocation of mitochondrial proteins fail, these precursor proteins can be degraded by ERAD or MAD.^364^ Both ERAD and MAD dynamically regulate the protein level of VISA, which is primarily localized at MAMs, thereby ensuring the innate immune response before and after viral infection.^170^ Moreover, the central components of MAD are shared with ERAD. ERAD and MAD share the Cdc48/p97/VCP ATPase and the adaptor proteins Ubx2 and Doa1/Ufd3, which facilitate the interaction of ubiquitinated substrates with Cdc48/p97/VCP.^365–368^ Ubx2, the ER-embedded ERAD component, assembles into a cluster on the outer mitochondrial membrane and engages Cdc48 to maintain the functional integrity of the translocase of the outer membrane (TOM) complex by preventing its obstruction upon the accumulation of translocation intermediates.^366,367,369^ In addition to Cdc48, Msp1 in yeast (ATAD1 in mammals) facilitates the extraction and subsequent degradation of mitochondrial proteins. Msp1 was originally identified as the dislocase for peroxisomal and ER tail-anchored proteins that are aberrantly targeted and incorporated into the mitochondrial outer membrane.^370,371^ Mistargeted tail-anchored proteins are extracted by Msp1 and then targeted to the ER surface, where the proteins are ubiquitinated by Doa10 and degraded by ERAD in a Cdc48–Ufd1–Npl4-dependent manner.^339^ Interestingly, Msp1 is a target for MAD, whose degradation depends on the Doa1–Cdc48 complex.^368^ Overall, the crosstalk between ERAD and MAD is important for mitochondrial quality control and homeostasis.

Over the past decade, accumulating evidence has demonstrated that ER–Golgi crosstalk is a key regulatory axis that governs cell communication and mediates both physiological and pathological responses.^372^ ER–Golgi juxtaposition provides a platform where bidirectional lipid trafficking and protein secretion are orchestrated. The contact sites between the ER and Golgi membrane harbor not only the pleckstrin homology (PH) domain and diphenylalanine (FF) in an acidic tract (FFAT) motif, which function in tethering the two membranes, but also the oxysterol-binding protein (OSBP)-related domain (ORD), which allows sterols to be transferred bidirectionally.^373^ Over the years, independent studies have addressed the fate of damaged proteins that evade the ER and how post-ER quality control at the Golgi is regulated. At the contact sites between the ER and Golgi, the Golgi apparatus can bring misfolded proteins back to the ER, whereas the ER possesses the capacity to differentiate properly folded proteins from incompletely folded intermediates that necessitate further folding assistance from chaperones, as discussed earlier.^374^ MHC proteins are retrieved to the ER from the cis-Golgi and degraded by ERAD.^375,376^ Retrieval to the ER for ERAD requires substrate recognition by retention in endoplasmic reticulum sorting receptor 1 (Rer1)^377^ or surfeit locus protein 4 (Surf4),^378,379^ both of which reside in the cis-Golgi. Notably, endosome and Golgi-associated degradation (EGAD) functions in safeguarding the ER against the improper accumulation of proteins.^110,380^ EGAD represents a cellular pathway responsible for the targeted degradation of integral membrane proteins via the proteasome and cooperates with endosomal sorting complexes required for transport (ESCRT) and ERAD. Golgi membrane proteins are targeted for proteasomal degradation via EGAD without being retrieved to the ER.^380^ The E3 ubiquitin ligase Dsc, which has been shown to direct SREBP to proteasomal degradation through an ERAD-independent mechanism in yeast, is implicated in EGAD.^381,382^ The ER-resident membrane protein orosomucoid 2 (Orm2) in yeast has been identified as another substrate for EGAD.^380^ Orm2 is ubiquitinated, extracted from the ER membrane via the Cdc48/p97/VCP ATPase, and targeted for proteasomal degradation. However, the substrates of EGAD and the existence of homologous mechanisms in mammals have yet to be elucidated. Furthermore, future work should address the determinants that distinguish proteins targeted to ERAD in the ER from those requiring first trafficking to the Golgi apparatus.

Endosomes transport proteins between the ER and the Golgi apparatus. Endosomes acquire MCSs with the ER early in their maturation, such that most early and all late endosomes are tethered to the ER.^383^ MCSs are important for lipid homeostasis, cargo sorting within endosomes, endosome trafficking, and endosome fission.^352^ A number of components control endosome organization and function. These proteins include the E3 ubiquitin ligase RNF26, which forms a complex with several ER cofactors, including TMEM43, endonuclease domain-containing protein 1 (ENDOD1), TMEM33, and transmembrane emp24 domain-containing protein (TMED1). These complexes allow RNF26 control of the perinuclear positioning of endolysosomes, which restrains fast transport of diverse vesicles, spatiotemporally orchestrating endosomal maturation and cargo trafficking.^108^ RNF26 also interacts with UBE2J1 to ubiquitinate p62, which in turn recruits endosomal adaptors to facilitate trafficking of activated epidermal growth factor receptor (EGFR) to lysosomes, a mechanism implicated in the control of EGF-induced AKT signaling.^109^ Early and late endosome shrinkage and fission are among the factors that control the trafficking of cargo from late endosomes to the Golgi, which is spatiotemporally associated with ER contact sites.^384^ The transmembrane and coil domain family 1 (TMCC1) and Coronin 1 C are located at the MCS and control the process of endosomal fission. TMCC1 functions to stabilize the MCS, and its depletion leads to defective cargo trafficking from late endosomes to the Golgi apparatus.^385^ The MCS also plays a role in preventing misfolded proteins from being delivered to lysosomes via secretory trafficking pathways, as exemplified by the ability to trap misfolded mucin 1 (MUC1) in TMED9 cargo receptor-containing early endosomes between the ER and Golgi apparatus.^386^

The ER–lysosome MCS, an essential coordinator of membrane trafficking, is critical for regulating cellular processes associated with the ER and lysosomes. These include lysosomal damage repair, lysosome positioning and motility, organelle dynamics, cellular metabolism, and signal transduction.^352,353,387,388^ The same proteins are engaged in ER-lysosome and ER-endosome MCSs despite their functional and compositional differences.^389^ Oxysterol-binding protein-related protein 1 L (ORP1L) resides in late endosomes/lysosomes and is recognized by vesicle-associated membrane protein-associated protein A (VAP-A), which is localized at the ER membrane, facilitating the juxtaposition of the lysosome and the ER.^390^ The juxtaposition of lysosomes with the ER promotes the translocation of a broad spectrum of proteins into the ER membrane.^391^ Importantly, for ER tubule elongation and connection, lysosomes are anchored to ER growth tips, indicating the involvement of ER–lysosome MCSs in ER expansion and remodeling.^392^ ER–lysosome MCSs are also engaged in the exchange of Ca^2+^. ER–lysosome MCSs mediate the establishment of physical and functional coupling between the ER and lysosome as crucial intracellular Ca^2+^ storage sites and stimulate adequate Ca^2+^ signaling.^393^ Lysosomal Ca^2+^ signaling can be amplified by IP_3_Rs or RyRs in tightly apposed ER regions.^394^ IP_3_Rs assemble into clusters or puncta on the ER membrane and preferentially interact with lysosomes to mediate the selective transport of Ca^2+^ from the ER to lysosomes.^395^ Ca^2+^ signals mediated by IP_3_Rs are rapidly transferred to lysosomes and govern processes such as endocytic fusion and fission, autophagy, and lysosomal biogenesis.^396,397^ Notably, interactions between the ER and lysosomes also exert regulatory effects on lysosomal network dynamics. Depletion of ER-shaping proteins such as reticulon attenuates the dynamic processes of lysosomal fission and fusion.^398^ A key future direction is to determine how ERAD influences the regulation of ER–lysosome MCS formation and their various functions.

Lipid droplets serve as storage organelles that are composed of a hydrophobic core filled with stored neutral lipids. Lipid droplets are encapsulated by a unique phospholipid bilayer that is often continuous with the outer leaflet of the ER membrane, which sets them apart from canonical MCSs.^399^ Noncanonical membrane bridges allow protein trafficking between lipid droplets and the ER.^352^ The formation of such lipid droplets is induced upon ER stress.^400^ This process requires free fatty acid esterification into triglycerides, which results in the formation of lipid droplets, enabling the mitigation of lipotoxic ER stress^401^ by clearing misfolded protein in inclusion bodies^402^ for ApoB100 and HMGCR degradation,^403^ suggesting that they function in the interplay between proteotoxicity and lipotoxicity.

### Linking ERAD to proteasome homeostasis

The ability of ERAD to elicit its ability to control protein well-being tightly depends on the proteasome, which serves as a clearance site for misfolded proteins extracted from the ER. The key to maintain proteasome biogenesis is the adaptive proteotoxic stress response, which is controlled by Nrf1.^404–406^ Nrf1 possesses an N-terminal TMD that targets it to the ER and a C-terminal DNA-binding domain.^407,408^ Upon *N*-linked glycosylation in the ER, Nrf1 is subjected to ERAD via its recognition by HRD1.^409^ Under conditions of low proteasome activity, Nrf1 is translocated from the ER into the nucleus. Inhibition of proteasome activity results in ubiquitination-dependent proteolytic cleavage of Nrf1 by aspartyl protease DNA damage inducible 1 homolog 2 (DDI2), which removes ~100 amino acids from the N-terminal TMD, resulting in Nrf1 activation with the subsequent upregulation of genes required for proteasome biogenesis.^410^ Notably, DDI2 recognition and processing of Nrf1 require long polyubiquitin chains, unlike those normally required for proteasomal degradation.^410^ In *Caenorhabditis elegans*, the accumulation of proteins with a propensity to misfold and aggregate triggers the upregulation of genes required for proteasome biogenesis by the Nrf1 ortholog SKN-1A.^411^ Impaired Nrf1 activity in bone, adipocytes, liver, or brain causes tissue development abnormalities.^334,412^ Nrf1 inactivation in the mouse brain promotes ubiquitinated protein aggregation and neurodegeneration,^412^ suggesting that proteasome homeostasis governed by Nrf1 as part of ERAD plays a role in normal physiology and development.

## Erad and disease

**Fig. 7:**
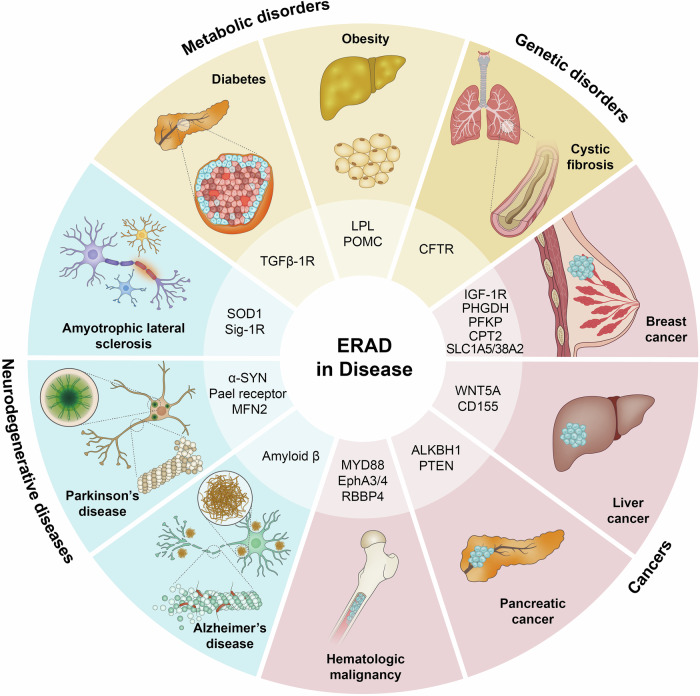
ERAD in diseases. ERAD has been linked to a wide range of diseases, including various cancers; neurodegenerative disorders, such as amyotrophic lateral sclerosis (ALS), Parkinson’s disease and Alzheimer’s disease; metabolic diseases, such as obesity and diabetes; and genetic disorders, such as cystic fibrosis. Proteins associated with diseases are also described (see the text for details)

The ER is important for cellular health in eukaryotes, as it serves as a central hub to secure the correct folding and maturation of proteins essential for diverse physiological processes, including growth, metabolic function, overall immune regulation, and behavior.^49,159^ Disease-associated mutant proteins are linked with ER retention, misfolding, and aggregation, similar to the accumulation of mutant proteins.^8^ Among disease-associated mutants are proinsulin, which is implicated in the syndrome of mutant INS gene-induced diabetes of youth (MIDY),^413^ POMC, which is involved in early-onset obesity,^414^ proAVP, which is involved in the autosomal dominant form of diabetes insipidus,^415^ and thyroglobulin, which is involved in congenital hypothyroidism.^416^ As discussed above, ERAD plays a crucial role in maintaining both the quality and quantity of proteins at the ER and is tightly interconnected with the UPR, ER-phagy, and other cellular stress programs. ERAD elicits diverse functions by impacting gene transcription and protein translation. When impaired, ERAD is associated with the development of various diseases, such as cancer, metabolic and genetic diseases, and neurodegenerative disorders (Fig. 7).^9,10,160,211^ Indeed, mutations in genes encoding ERAD components have been associated with disease pathogenesis,^9^ suggesting that ERAD is involved in disease progression.

### ERAD and metabolic disorders

The development and analysis of cellular and mouse models with cell-type-specific deficiency of the HRD1 complex have contributed greatly to clarifying the role of ERAD in energy metabolism, water balance, immune responses, and aging.^8,10,157,159,224,227,276,277,417^ For example, to maintain systemic water balance, misfolded AVP is degraded by the HRD1 complex.^277^ The HRD1 complex is also involved in insulin content via its activities in β cells. By targeting transforming growth factor beta (TGF-β) receptor I for ERAD, HRD1 controls β-cell identity.^418^ The total insulin content increases upon inhibition of TGF-β signaling in SEL1L-deficient β cells, providing a framework for therapies targeting ER PQC. Adipocyte-specific SEL1L depletion promotes the aggregation and accumulation of LPL in the ER, leading to postprandial hypertriglyceridemia in mice.^419^ In the mouse liver, HRD1-mediated ERAD is constitutively active under basal conditions.^417,420^ HRD1 facilitates proteasomal degradation of the liver-specific ER-tethered transcription factor cAMP-responsive element-binding protein (CREBH) to downregulate fibroblast growth factor 21 (FGF21) expression. HRD1 directs a fraction of nascent POMC to the ERAD and prevents POMC aggregate accumulation, ensuring that other POMC fractions undergo proper posttranslational maturation and transport for secretion.^276^ Mice with POMC neuron-specific SEL1L depletion develop age-associated obesity and hyperphagia owing to POMC retention within the ER, suggesting that HRD1 complex-mediated ERAD functions in the development of monogenic obesity associated with impaired prohormone folding.^276,421^ Taken together, these observations identify the important role of ERAD, through its diverse components exemplified by HRD1 here, in the control of metabolic networks, from lipid metabolism to insulin tolerance, which are implicated in a number of physiological processes, from aging to obesity.

### ERAD and CFTR

Protein misfolding is implicated in several diseases, many of which are genetically determined. Wild-type proteins are synthesized, integrated into the ER membrane, and subsequently translocated to the cell surface, usually via the secretory route. In contrast, misfolded mutant proteins cannot reach the cell surface and instead accumulate in the ER, promoting ER stress.^9^ CFTR is commonly mutated among patients suffering from cystic fibrosis, with F508 deletion (F508del), the most common hotspot mutation.^119^ CFTR is a 168 kDa protein and a member of the ATP-binding cassette transporter family, and its ATP-binding capacity allows it to facilitate the translocation of substrates across cellular membranes. However, CFTR functions as a channel, not a transporter, using ATP to shift between the open and closed conformations. As a result, in epithelial cells of the lung or digestive tract, where it is commonly expressed, CFTR activity ensures proper chloride and bicarbonate transport out of cells into the extracellular space. While the export of chloride results in the concomitant transport of water, the export of bicarbonate alters acidity, and both outcomes cause chronic clinical manifestations in patients with mutant CFTR. CFTRs are folded by HSP70 family proteins,^422^ which often recruit other chaperones (i.e., HSP90). F508del CFTR, similar to other mutant forms of CFTR, is held within the ER and directed to the ERAD, where they engage E3 ubiquitin ligases, including CHIP and RNF5, which are implicated in recognition, ubiquitination, and clearance.^119,146,423^ More recent studies suggest that mutant CFTR is subject to increased endocytosis and lysosomal degradation,^424^ highlighting additional organelles that are affected and thus have more complex physiological consequences.

Pharmacological modulators are available to target specific mutant forms of CFTR, which are designed to enable refolding and thus to restore some of its function, including its ability to reach the cell surface.^425^ In addition, strategies for the evasion of mutant CFTR from ERAD and its trafficking to the cell surface have been suggested to have therapeutic potential.^426^ CFTR contains two TMDs and two nucleotide-binding domains (NBDs). CFTR normally folds in a way that conceals four partially redundant arginine-framed tripeptide (AFT) sequences located within NBD1 of CFTR; if exposed to the cytoplasm, these sequences promote CFTR retention within the ER.^427^ Mutation of the AFT sequence results in the trafficking of F508del CFTR and ensures that CFTR functions at the cell surface, although the majority of F508del CFTR retention and degradation cannot be attributed solely to AFT sequences.^427^ Intriguingly, some studies indicate that F508del CFTR is capable of bypassing the conventional ER–Golgi-mediated trafficking pathway and instead utilizes an unconventional protein secretion pathway to reach the plasma membrane.^428–430^ The unconventional protein secretion pathway requires Golgi reassembly stacking protein (GRASP) and vesicular components linked to secretory autophagy, a nondegradative autophagic process promoting the trafficking of cargo to the plasma membrane.^429^ Mammalian autophagic components implicated in this pathway include gamma-aminobutyric acid receptor-associated protein (GABARAP), Unc-51-like kinases (ULKs), Beclin, and LC3.^431^ The unconventional protein secretion pathway is triggered by cellular stresses, such as inflammation and ER stress, which activate the UPR. Disruption of ER-to-Golgi trafficking or ER stress promotes F508del CFTR transport to the plasma membrane through Golgi-bypassing secretory autophagy in a GRASP55-dependent manner.^426,432^ Notably, the F508del CFTR delivered through the unconventional secretion pathway exhibits sufficient functionality,^429^ suggesting that restraining ERAD and promoting trafficking of mutant forms of CFTR to the cell surface represent another therapeutic intervention targeting cystic fibrosis.

### ERAD and neurodegenerative diseases

Proteotoxicity has also been associated with progressive neuronal damage. The ER PQC pathways have been demonstrated to function in mitigating proteotoxicity in neurons. Moreover, over the past decade, numerous studies have demonstrated the critical roles of ERAD in the development of neurodegenerative diseases. Dysregulation of ERAD components, including ERLIN, MBRL, SEL1L, HRD1, and Derlin, has been shown to play roles in neuronal impairment and death in animal models of neurodegenerative diseases.^433,434^ Deficiency of SEL1L within Purkinje cells causes early-onset, progressive cerebellar ataxia accompanied by age-dependent progressive loss of Purkinje cells.^435^ Patients carrying a biallelic SEL1L variant (p.Cys141Tyr) exhibit both ERAD-associated neurodevelopmental disorders with onset in infancy (ENDI) syndrome and infantile-onset agammaglobulinemia with no mature B cells, leading to recurrent infections and early mortality.^436^ This variant causes severe SEL1L–HRD1 ERAD dysfunction via disruption of disulfide bond formation within the luminal fibronectin II domain of SEL1L and subsequently causes SEL1L degradation by HRD1. Furthermore, in six children from three unrelated families, biallelic missense variants of SEL1L and HRD1 have been linked to ataxia, facial dysmorphisms, microcephaly, developmental delay, hypotonia, and/or intellectual disability.^437^ Variants in HRD1 (p. Pro398Leu) and SEL1L (p. Gly585Asp and p. Met528Arg) impair the multistep ERAD process, including substrate recruitment, SEL1L-HRD1 complex formation, and HRD1 enzymatic activity.

The most common lysosomal storage disorder (LSD), Gaucher disease (GD), is an autosomal recessive sphingolipidosis resulting from mutations in a lysosomal enzyme, glucocerebrosidase (GCase), or its activator protein, saposin C, which mediate the hydrolysis of glucosylceramide (GlcCer) into ceramide and glucose.^438^ Thus, GD is defined by GlcCer accumulation. Mutant GCase proteins are improperly folded, retained within the ER, and inefficiently trafficked to lysosomes, instead being targeted for ERAD.^439^ Notably, the degree of ER retention and subsequent ERAD of GCase mutants differs, and this variation is strongly linked to GD severity.^440^

A progressive neurological disorder, Alzheimer’s disease (AD), is defined by cognitive impairment and behavioral disturbances.^441^ The aggregation of amyloid β peptides and the formation of neurofibrillary tangles, known as intracellular aggregates of hyperphosphorylated tau protein, represent the main features contributing to the pathogenesis of AD. Proteolytic cleavage of amyloid precursor protein (APP) to generate amyloid β is one of the key characteristics of AD.^442^ Interestingly, RHBDL4 has been shown to cleave APP in its ectodomain, which reduces amyloid β peptide production.^443^ Altered MAM function has also been linked to AD pathogenesis.^296,444^ APP cleavage and amyloid β peptide production partially occur at MAMs.^445^ The ER transmembrane proteins presenilins (PS1) 1 and 2, key components of APP processing, are detected in MAMs.^445,446^ PS1 and PS2 mutations are implicated in the disruption of calcium homeostasis, elevated production of amyloid β, and increased susceptibility to ER stress–induced apoptosis and are responsible for most familial forms of AD.^447^ Notably, Nrf1 is downregulated in AD.^448^ A brain-specific knockout of *Nrf1* results in proteasome impairment and neuronal apoptosis.^412^ In *Caenorhabditis elegans*, SKN-1A/Nrf1 attenuates amyloid β accumulation and delays adult-onset cellular dysfunction. In addition, Nrf1 upregulates HRD1 to attenuate the ER stress-induced apoptosis of neuroplastic cells.^449^ Elucidating the binding activity of Nrf1 to the *Hrd1* promoter in AD patient–derived neurons could be highly interesting.

Parkinson’s disease (PD) represents another progressive neurological disorder characterized by severe oxidative stress, mitochondrial impairment, and the accumulation of misfolded α-synuclein (α-SYN) that aggregates into proteinaceous inclusions in Lewy bodies (LBs) or Lewy neurites.^450^ α-SYN aggregates bind to BiP, which in turn activates the UPR.^451^ In addition, α-SYN aggregates activate SERCA in neurons, thereby altering cell death, ROS production, and calcium metabolism.^452^ Notably, the aggregation of ubiquitin conjugates in neurons frequently impairs proteasome activity, thereby further promoting α-SYN aggregate accumulation and subsequent neuronal injury, which drives the development of PD.^453^ Interestingly, Nrf1 is downregulated in PD, suggesting that Nrf1 upregulation may alleviate impaired protein turnover, thereby reducing proteotoxicity and enhancing neuronal resilience against PD pathogenesis.^453,454^ As an E3 ubiquitin ligase, PARKIN functions in a wide range of cellular processes associated with PD.^455,456^ The upregulation of PARKIN in response to ER stress functions to protect neurons from ER stress.^456^ The Pael receptor, which is implicated in ER stress–driven cell death, serves as a substrate of PARKIN.^457^ The Pael receptor can occasionally misfold and form aggregates, suggesting that Pael receptor degradation by PARKIN might protect dopaminergic neurons against diverse forms of damage and attenuate the toxicity driven by the Pael receptor. Moreover, in Drosophila models, PARKIN overexpression leads to increased K48-linked polyubiquitination and reduced protein aggregation.^458^ PARKIN is found at MAMs, where it may facilitate MFN2 ubiquitination and degradation.^459^ Similarly, leucine-rich repeat serine/threonine-protein kinase 2 (LRRK2), a protein linked to PD, localizes to MAMs, where it enhances PERK-mediated phosphorylation of PARKIN, MARCHF5, and mitochondrial E3 ubiquitin ligase activator of NF-κB (MULAN), thereby facilitating the ubiquitination of MFN2.^460^

The most prevalent adult-onset motor neuron degeneration, amyotrophic lateral sclerosis (ALS), impacts both upper and lower motor neurons in the cortex, brainstem, and spinal cord, leading to gradual wasting and paralysis of voluntary muscles.^461^ SOD1 is mutated in certain cases of familial ALS (FALS), which is an inherited subtype of the illness.^462^ Mutant forms of SOD1, including G85R-SOD1, G37R-SOD1, or G93A-SOD1, might have acquired unfavorable features that drive the development of FALS.^463^ In the spinal cords of transgenic ALS mice harboring a mutant form of SOD1 and in human ALS spinal cord samples, SOD1 aggregates have been shown to accumulate,^463^ suggesting that mutant SOD1 aggregate accumulation could be associated with adverse effects within motor neurons in individuals with ALS, although it cannot be considered sufficient to account for the development of ALS. Accumulating evidence highlights that MAMs are associated with the pathological relevance observed in both FALS and sporadic ALS, such as mitochondrial impairment, disrupted Ca^2+^ homeostasis, improper protein folding, and the ER stress response.^464^ Downregulation of Sig-1R, the major determinant of MAM structure, has been observed in lumbar spinal cord specimens derived from sporadic ALS patients, together with an increase in BiP.^465^ In addition, Sig-1R depletion induces the accumulation of misfolded proteins.^465^ Notably, the mutant form of Sig-1R (E102Q) has been shown to be associated with its aggregation and accumulation in the ER.^466^ In line with this, lymphoblastoid cells derived from FALS patients harboring the mutant form of Sig-1R (E102Q) exhibit elevated ER stress.

Overall, these studies suggest crucial roles for ERAD in neurodegenerative diseases, including AD, PD, and ALS. Therefore, the role of ERAD in the pathophysiological progression of neurodegenerative diseases needs to be further thoroughly addressed.

### ERAD and cancer pathogenesis

**Fig. 8:**
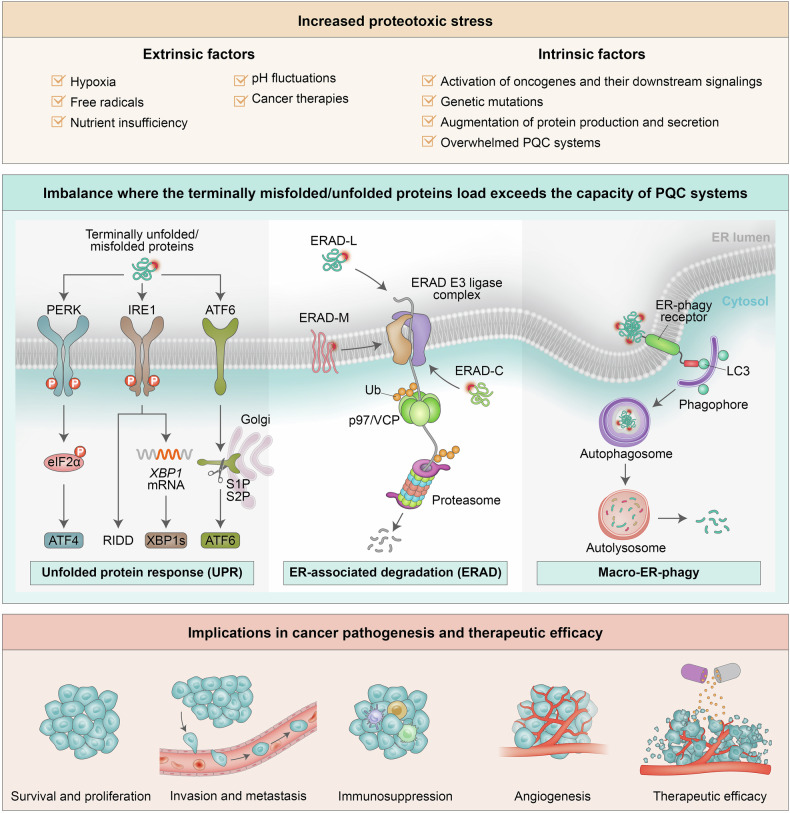
ER protein quality control (PQC) systems and cancer. In cancer cells, proteotoxic stress is increased and sustained by both extrinsic factors, including hypoxia, free radicals, nutrient insufficiency, pH fluctuations, and various cancer therapies, and intrinsic factors, including the activation of oncogenes and downstream oncogenic signaling, genetic mutation, increased protein production and secretion, and overload of PQC systems. These conditions result in an imbalance in which the terminally misfolded/unfolded protein load exceeds the capacity of PQC systems in the ER. These conditions are associated with cancer pathogenesis and impact therapeutic efficacy

Cancer cells are continuously exposed to environmental stress, such as low oxygen levels (hypoxia), nutrient insufficiency, pH fluctuations, and anticancer radiation treatment and/or chemotherapy. All of these activities alter cellular signaling, impact protein production to overwhelm PQC, and perturb ER functions, leading to proteotoxicity (Fig. 8).^467,468^ Therefore, cancer cells require high proteostatic capacity to counteract constant proteotoxic stress. ERAD dysregulation is implicated in cancer development, progression, and resistance to therapy,^469,470^ although altering ERAD to overcome these conditions has not yet been thoroughly investigated.

In breast cancer and possibly other tumor types, HRD1 reportedly mediates ERAD of insulin-like growth factor-1 (IGF-1R),^471^ the platelet isoform of phosphofructokinase (PFKP),^472^ and carnitine palmitoyltransferase 2 (CPT2),^473^ which are key pathways involved in breast cancer pathogenesis. Similarly, estrogen receptor β 1 (ERβ1) promotes HRD1-mediated ERAD of IRE1α, which attenuates the ER stress-induced IRE1α-XBP1 pathway in breast cancer.^474^ RNF5 ubiquitinates and promotes the degradation of phosphoglycerate dehydrogenase (PHGDH), the rate-limiting enzyme in serine synthesis and a factor upregulated in human breast cancer, attenuating breast cancer cell proliferation.^475^

HRD1 upregulation is associated with the propensity of hepatocellular carcinoma (HCC) to metastasize.^476^ The upregulation of the SEL1L-HRD1 complex, which is positively correlated with survival time in liver cancer patients, promotes ERAD of misfolded WNT5A.^477^ IRE1α contributes to HRD1 upregulation in HCC cells, leading to CD155 degradation.^478^ HRD1 has also been implicated in mitochondrial biogenesis. Increased HRD1 activity, upon SIRT4-mediated SEL1L deacetylation, decreases the stability of mitochondrial protein alkylation B homolog 1 (ALKBH1), inducing mitochondrial damage in pancreatic ductal adenocarcinoma (PDAC).^479^ Treatment with entinostat, a SIRT4 stimulator, inhibits pancreatic cancer in vivo and in vitro.^479,480^ The myeloid-specific HRD1 complex has been implicated in the ubiquitination of STING, resulting in its degradation in the basal state and limiting its role in antitumor immunity.^481^

RNF5 was shown to promote the degradation of misfolded SLC1A5 and SLC38A2 after paclitaxel treatment of breast cancer cells, which resulted in decreased glutamine uptake and increased apoptosis of breast cancer cells.^176^ Mice lacking RNF5 exhibit attenuated UPR activation, which coincides with the upregulation of inflammasome components, concomitant with the downregulation of antimicrobial peptides in intestinal epithelial cells.^482^ In lymphoma cells, RNF5 selectively targets the myeloid differentiation primary response protein 88 (MYD88) mutant (L265P), which is common in patients with hematological malignancies, for degradation, likely due to its misfolding.^483^ In primary effusion lymphoma cells, RNF5 has been implicated in the control of ERK and AKT signaling through its regulation of Ephrin receptors A3 (EphA3) and EphA4, a control that inhibits xenograft tumor growth.^484^

RNF5 induces the formation of noncanonical Lys29 ubiquitin chains on retinoblastoma binding protein 4 (RBBP4), which results in the recruitment of RBBP4 to histones, where it alters the transcription of genes that are important for acute myeloid leukemia (AML).^485^ Similarly, the development of MLL-AF9-driven leukemogenesis is attenuated in *Rnf5*-KO mice.^485^ The low stromal levels of phosphatase and tensin homolog (PTEN) in PDAC patients can also be attributed to RNF5 function, as RNF5 was shown to catalyze the degradation of PTEN.^486^ The depletion of RNF5, or pharmacological blockade of glycogen synthase kinase 3β (GSKβ), rescues PTEN levels and reverses oncogenic phenotypes in the PDAC stroma.

These studies point to tissue-specific targets of RNF5, suggesting that substrate recognition as part of ERAD function differs, likely because of tissue-specific expression or posttranslational modification.

## Erad and therapeutics

Recently, the field of proteostasis-based therapy has significantly advanced, including the utilization of HSP inhibitors, proteasome and autophagy inhibitors, and UPR and ERAD inhibitors.^487^ Among the key players of ERAD, HRD1 has been suggested to be a target for drug development.^8,488^ HRD1 expression is elevated in the rheumatoid arthritis synovium and is implicated in the development of rheumatoid arthritis.^489^ In addition, HRD1 promotes IRE1 ubiquitination and degradation, which is associated with the apoptosis of synovial cells upon ER stress.^490^ Notably, LS-101 and LS-102 function as enzymatic inhibitors that target HRD1, and among them, LS-102 selectively targets HRD1 and markedly attenuates the severity of rheumatoid arthritis in in vivo models.^491^ ERAD mediated by HRD1 has been shown to be a crucial factor for flavivirus replication.^492^ Interestingly, CP26, a small molecule inhibitor of substrate translocation from the ER to the cytoplasm during ERAD, targets the SEL1L-HRD1 complex and attenuates SEL1L-HRD1 ERAD, thereby inducing ER stress, which results in substantial protection of host cells against virus-induced cell death.^493^

Inhibitors of p97/VCP ATPase include eeyarestatin I (EerI), DbeQ, ML240, ML241, NMS-873, and CB-5083.^494–497^ EerI attenuates ERAD and activates ATF3 and ATF4, resulting in the upregulation of proapoptotic NOXA in multiple myeloma and mantle cell lymphoma.^498^ Immunogenic cell death (ICD) plays a pivotal role in eliciting anticancer immune responses.^499,500^ ICD enables tumors to trigger dendritic cell maturation and the activation of adaptive immunity mediated by cytotoxic T cells.^499^ The immunogenicity of esophageal cancer is stimulated by a moderate dose of radiotherapy, which is significantly enhanced by the combination of NMS-873 and EerI.^501^ Salirasib, also referred to as farnesyl thiosalicylic acid (FTS), functions as a RAS inhibitor in PDAC cells by specifically dislodging active RAS proteins from the plasma membrane, suppressing their downstream signal transduction, and inducing UPR activation, which is further augmented upon EerI treatment.^502^ Importantly, the combined treatment of FTS with EerI substantially induced apoptosis in pancreatic cancer cells. CB-5083 activates the UPR and induces apoptotic cell death in a broad spectrum of hematological and solid tumors *both* in vivo and in vitro.^503,504^

Notably, oncogenic and tumor suppressor proteins are present at MAMs, and their alterations affect Ca^2+^ flux, lipid homeostasis, and mitochondrial dynamics. Therefore, their dysregulation at MAMs might influence the malignant characteristics of cancer cells, including excessive proliferation, apoptosis evasion, and metabolic reprogramming, suggesting that MAM targeting could be a new potential therapeutic strategy in cancer treatment. F-box protein F-box/LRR-repeat protein 2 (FBXL2), a receptor subunit of SCF (SKP1, CUL1, F-box protein) E3 ubiquitin ligase complexes, binds to IP_3_R3 and promotes IP_3_R3 ubiquitination and degradation, limiting ER-to-mitochondria Ca^2+^ transfer and attenuating apoptosis.^505^ PTEN antagonizes FBXL2 binding to IP_3_R3, thereby maintaining IP_3_R3 stability. The nondegradable mutant form of IP_3_R3 enhances the susceptibility of PTEN-deficient or low-PTEN tumor cells to photodynamic therapy through photosensitizer drug-induced Ca²⁺-mediated cytotoxicity, suggesting that in PTEN-deregulated cancers, preventing the degradation of IP_3_R3 constitutes a promising therapeutic approach.

The development and approval of proteasome inhibitors have provided therapeutic options for treating malignancies. Notably, proteasomes possess heterogeneity. The constitutive 26S proteasome is expressed in nearly all tissues and cell types and is composed of a 19S regulatory particle harboring ubiquitin receptors and an ATPase ring for protein unfolding and a 20S proteolytic core particle.^506^ The 20S core particle comprises four stacked rings, with each ring containing seven subunits. The inner two rings of the 20S core particle include β-subunits, which exhibit proteolytic activities due to their β1, β2, and β5 subunits, which possess caspase-, trypsin-, and chymotrypsin-like activities, respectively.^35,507^ Notably, in addition to the constitutive 26S proteasome, three other proteasome isoforms, including the spermatoproteasome, thymoproteasome, and immunoproteasome, exhibit tissue-specific expression.^508,509^ Among them, the immunoproteasome is constitutively expressed in cells of hematopoietic origin and can also be upregulated by cytokine stimulation in a variety of nonhematopoietic cell types as well as in hematopoietic cells.^510^ The immunoproteasome harbors immunosubunits β1i, β2i, and β5i instead of the β1, β2, and β5 subunits.^511–513^ The immunoproteasome plays a regulatory role in MHC class I antigen processing^514^ and is involved in T-helper cell differentiation.^515^ The immunoproteasome assembles four times faster than the constitutive 26S proteasome does in response to immune and inflammatory stimuli.^509,516^ Proteasome inhibition can, in principle, target any of the three proteolytic sites of the proteasome, β1, β2, and β5, and the majority of proteasome inhibitors developed to date target the β5/β5i subunits. Not surprisingly, the effectiveness of proteasome inhibitors has been well known for therapeutic intervention in multiple myeloma. As a hematological malignancy, multiple myeloma is characterized by clonal expansion of plasma cells within the bone marrow, which results in excessive production of monoclonal immunoglobulins.^517^ Plasma cells heavily depend on the ERAD for monitoring the quality and quantity of immunoglobulins synthesized in the ER.^157,518–520^ The ubiquitin E3 ligases UBR4 and UBR5 are associated with the ERAD of immunoglobulins.^518^ In addition, the SEL1L–HRD1 complex contributes to the degradation of immunoglobulins.^86,521^ Bortezomib, the first proteasome inhibitor approved by the Food and Drug Administration (FDA), directly targets the proteasome and promotes cell death.^522,523^ Bortezomib triggers ER stress and is widely recognized as an anticancer drug used to treat multiple myeloma and lymphoma.^524–526^ In addition to multiple myeloma types, bortezomib has shown cytotoxic activity across multiple malignancies, such as colon, breast, lung, and prostate cancer cells.^527,528^ Bortezomib treatment reduces microvessel density in multiple myeloma patients, which is positively associated with improved prognosis,^529^ indicating that antiangiogenic activity may serve as a prognostic marker for assessing the therapeutic outcomes of bortezomib. Notably, the use of bortezomib in combination with targeted therapies is considered a potentially effective approach for cancer treatment. Bortezomib in combination with the autophagy inhibitor hydroxychloroquine has been reported to be effective in treating refractory and relapsed multiple myeloma.^530^ Bortezomib in conjunction with the histone deacetylase (HDAC) inhibitor panobinostat, monoclonal antibodies such as elotuzumab and daratumumab, or the BCL-2 inhibitor venetoclax, yields synergistic outcomes in refractory and relapsed multiple myeloma.^531–534^ However, although bortezomib has achieved clinical success, its use has been associated with toxicity and the emergence of drug resistance.^535,536^ Second-generation proteasome inhibitors, including carfilzomib, marizomib, and ixazomib, which may overcome the resistance and toxicity of bortezomib, are being evaluated in clinical investigations for myeloma bone disease, chronic lymphocytic lymphoma, and multiple myeloma.^528,537–539^

Bortezomib, carfilzomib, and ixazomib indiscriminately target both the constitutive 26S proteasome and the immunoproteasome. This nonselective inhibition may partly explain the side effects common, as well as drug resistance relapse after prolonged treatment.^540^ Immunoproteasome inhibitors could overcome this nonselective inhibition, although they are still in the early stages of development for treating hematologic malignancies.

The development of inhibitors targeting the β5i subunit of the immunoproteasome has recently entered clinical trials. M3258, a reversible immunoproteasome β5i subunit inhibitor with oral bioavailability, was developed.^541^ M3258 treatment results in strong antitumor activity in multiple myeloma xenograft models and shows enhanced effectiveness relative to ixazomib or bortezomib. A phase I clinical trial was carried out to evaluate the tolerability of M3258 in patients with relapsed and refractory multiple myeloma.^542^ ONX-0914 represents the first selective immunoproteasome inhibitor designed to target the β5i subunit.^543^ ONX-0914 treatment is cytotoxic to multiple myeloma cells.^544^ Interferon-γ-induced upregulation of the immunoproteasome increases sensitivity to ONX-0914 but not to carfilzomib. In mice engrafted with xenografts derived from castration-resistant prostate cancer (CRPC) patients, treatment with ONX-0914 suppressed CRPC progression.^545^ ONX-0914 suppresses the proliferation of glioblastoma cells and induces apoptosis.^546^ Notably, ONX-0914 acts synergistically to improve the antitumor efficacy of ixazomib, carfilzomib, or bortezomib.^544^ Moreover, ONX-0914 and its derivatives have been reported to have therapeutic potential for a variety of inflammatory and autoimmune diseases, such as rheumatoid arthritis,^543^ multiple sclerosis,^547^ colitis,^548^ and lupus, and are currently under clinical evaluation.^549^

Taken together, these findings indicate that the modulation of ERAD holds significant promise as a new therapeutic frontier in the treatment of diseases. Undoubtedly, more sophisticated studies are needed to define the interaction network of ERAD during disease progression and to understand the effects of targeting ERAD while preserving the physiological functions of ERAD, which could help not only pave the way for new therapeutic strategies but also uncover potent and durable combination treatments.

## Conclusions and path forward

Proper integrity and functionality of the proteome, referred to as proteostasis, are essential for coordinating cellular demands and mitigating various intrinsic and extrinsic perturbations, including increased protein synthesis, ER–Ca^2+^ depletion, dysregulated redox homeostasis, energy deprivation, hypoglycemia, hypoxia, and inflammatory stimuli, thereby ensuring tissue integrity and organismal health.^1,2^ Undoubtedly, a fundamental challenge is deciphering how proteostasis is ensured in cellular organelles. The proteome of individual organelles is distinctive and energetic, constantly being optimally shaped and maintained by a number of PQC systems. The ER is a large and multifunctional organelle that is responsible for the biosynthesis, folding, maturation, stabilization, and trafficking of transmembrane and secretory proteins, accommodating approximately one-third of the proteome in eukaryotes. The ER also houses proteins and protein complexes essential for a variety of cellular functions, including growth, metabolism, stress responses, and immune responses. Furthermore, the ER is the main site for lipid biosynthesis and the storage of intracellular calcium. Given the essential roles of the ER, it is not surprising that the ER engages PQC systems, the ERAD, the UPR, and ER-phagy.

ERAD is the best-characterized PQC system of the ER. While ERAD is largely associated with the degradation of unwanted byproducts of protein biosynthesis, including misfolded and unassembled proteins, and safeguards protein maturation, it is now apparent that ERAD also regulates the abundance of folding-competent proteins as a means to fine-tune key physiological processes, suggesting that ERAD constitutively functions to ensure both protein quality and quantity.^18^ Therefore, ERAD plays key roles in the continuous shaping of the ER proteome, which consequently enables the ER to execute multiple functions, including lipid homeostasis, interorganelle contacts, calcium homeostasis, nuclear envelope organization, and the immune response.

The large repertoire of ERAD cellular functions includes substrate recognition, translocation, and ubiquitin processing. Although ERAD substrate delivery to the proteasome was discovered approximately three decades ago, recent studies have provided an advanced understanding of the mechanisms governing diverse ERAD regulators and their functions. By recognizing nascent polypeptides and targeting misfolded proteins, ERAD regulates activities as diverse as ribosome quality control or responses to cellular stress or lipid stress.^13^ In vitro reconstitution of ERAD processing will provide further valuable insights into the mechanism of ERAD. Moreover, structural refinement of ERAD complexes will be a major step forward in the field of the ER.

ERAD is tightly interconnected with the UPR, ER-phagy, and other cellular stress programs. ERAD elicits diverse functions by impacting gene transcription and protein translation. Thus, ERAD is expected to play a crucial role in the pathogenesis of various diseases, including cancer, metabolic and genetic disorders, neurodegenerative diseases, and therapeutics. Numerous stimuli activate ERAD—from genetic disorders that underlie the formation of misfolded proteins to somatic mutations that cause aberrant signaling and proteotoxic or lipotoxic stress—to environmental stress that drives epigenetic changes that alter protein conformation. Many of these stimuli also promote UPR signaling and ER-phagy to cope with cell stress and control cell death programs. Dysregulation of ERAD components has also been implicated in disease pathogenesis. Thus, an increased understanding of the fundamental pathways underlying ERAD regulation and function should encourage the development of therapies aimed at normalizing ERAD. For example, novel small molecules that redirect undesirable secretory proteins to the ERAD, attenuate the evasion of ERAD by disease-causing mutants, or inhibit pathogen-mediated hijacking of ERAD could serve as useful reagents for treating a plethora of diseases. Nevertheless, many questions remain concerning the basic biology of ERAD and its promising potential for medical intervention, including how equivalent ERAD substrates have evolved to require a broad range of E3 ubiquitin ligases provided by evolutionary diversification. How do the lipid environment or lipid composition select specific ERAD branches and determine the fate of ERAD substrates? How and why do ERAD E3 ubiquitin ligases spanning the ER membrane target transcription factors and cytosolic proteins for ERAD? How can the capacity and function of ERAD be accurately measured in a physiological or pathological context? Can the expression signatures of ERAD genes be used for the diagnosis and prognosis of disease pathogenesis? Can ERAD be targeted without altering upstream regulatory components, or can specific ERAD, ER-phagy, or UPR regulatory pathways be targeted to maximize selectivity? Among the challenging areas to be further investigated is how and when key PQC systems of the ER (ERAD, UPR, and ER-phagy) complement each other in a physiological or pathophysiological context. Addressing these questions will not only increase our understanding of ERAD but also contribute to the advancement of knowledge about its pathological implications. Moreover, additional insights should emerge from ongoing research and clinical evaluation of ERAD in the treatment of chronic diseases, including cancer.
